# Assembly of the Auditory Circuitry by a *Hox* Genetic Network in the Mouse Brainstem

**DOI:** 10.1371/journal.pgen.1003249

**Published:** 2013-02-07

**Authors:** Maria Di Bonito, Yuichi Narita, Bice Avallone, Luigi Sequino, Marta Mancuso, Gennaro Andolfi, Anna Maria Franzè, Luis Puelles, Filippo M. Rijli, Michèle Studer

**Affiliations:** 1 Telethon Institute of Genetics and Medicine (TIGEM), Naples, Italy; 2 Université de Nice-Sophia Antipolis, Nice, France; 3 INSERM UMR 1091, Nice, France; 4 Friedrich Miescher Institute for Biomedical Research, Basel, Switzerland; 5 Department of Biological Sciences, Università degli Studi di Napoli Federico II, Naples, Italy; 6 Institute of Audiology, University “Federico II”, Naples, Italy; 7 Institute of Genetics and Biophysics “A. Buzzati Traverso” C.N.R., Naples, Italy; 8 CEINGE Biotecnologie Avanzate, Naples, Italy; 9 Department of Human Anatomy and Psychobiology, University of Murcia, Murcia, Spain; 10 University of Basel, Basel, Switzerland; Samuel Lunenfeld Research Institute, Canada

## Abstract

Rhombomeres (r) contribute to brainstem auditory nuclei during development. *Hox* genes are determinants of rhombomere-derived fate and neuronal connectivity. Little is known about the contribution of individual rhombomeres and their associated *Hox* codes to auditory sensorimotor circuitry. Here, we show that r4 contributes to functionally linked sensory and motor components, including the ventral nucleus of lateral lemniscus, posterior ventral cochlear nuclei (VCN), and motor olivocochlear neurons. Assembly of the r4-derived auditory components is involved in sound perception and depends on regulatory interactions between *Hoxb1* and *Hoxb2*. Indeed, in *Hoxb1* and *Hoxb2* mutant mice the transmission of low-level auditory stimuli is lost, resulting in hearing impairments. On the other hand, *Hoxa2* regulates the *Rig1* axon guidance receptor and controls contralateral projections from the anterior VCN to the medial nucleus of the trapezoid body, a circuit involved in sound localization. Thus, individual rhombomeres and their associated *Hox* codes control the assembly of distinct functionally segregated sub-circuits in the developing auditory brainstem.

## Author Summary

Sound perception and sound localization are controlled by two distinct circuits in the central nervous system. However, the cellular and molecular determinants underlying their development are poorly understood. Here, we show that a spatially restricted region of the brainstem, the rhombomere 4, and two members of the *Hox* gene family, *Hoxb1* and *Hoxb2*, are directly implicated in the development of the circuit leading to sound perception and sound amplification. In the absence of Hoxb1 and Hoxb2 function, we found severe morphological defects in the hair cell population implicated in transducing the acoustic signal, leading ultimately to severe hearing impairments in adult mutant mice. In contrast, the expression in the cochlear nucleus of another *Hox* member, *Hoxa2*, regulates the guidance receptor *Rig1* and contralateral connectivity in the sound localization circuit. Some of the auditory dysfunctions described in our mouse models resemble pathological hearing conditions in humans, in which patients have an elevated hearing threshold sensitivity, as recorded in audiograms. Thus, this study provides mechanistic insight into the genetic and functional regulation of *Hox* genes during development and assembly of the auditory system.

## Introduction

The mammalian brainstem plays a crucial role in the regulation of many vital functions through a complex system of reflex arcs and relays information to higher brain centers through interconnected neuronal circuits. During development, the hindbrain becomes subdivided along the antero-posterior (A-P) axis into serially repeated, spatially segregated, modules of progenitor cells, the rhombomeres (r). Individual rhombomeres give rise to distinct portions of sensory and motor columns depending on the position of progenitors along the dorso-ventral (D-V) axis, respectively [1], thus generating nuclei of multi-segmental origin and topographic patterns of connectivity [2], [3], [4], [5], [6], [7]. For instance, in the somatosensory system, afferent innervation from mandibular or whisker pad (maxillary) facial dermatomes targets the r2- or r3-derived components of the principal trigeminal sensory nucleus, respectively. In turn, the information is somatotopically relayed to the thalamus and somatosensory cortex, contributing to build a facial somatosensory map [7]. Vestibular nuclei also originate from different rhombomeres and display specific sets of axonal trajectories with distinct targets [3], [4], [8], [9]. Thus, regional patterning along the A-P axis and specific D-V determinants intersect to determine sub-circuit connectivity within functionally related longitudinal neuronal columns.

Topographic connectivity and employment of sensory and motor nuclei are also well described during formation of auditory-dependent circuits [10]. The auditory central pathway consists of sensory nuclei transmitting ascending acoustic information, and efferent motor neurons modulating primary afferent responses. The sensory organ for sound is the cochlea, which contains two types of receptors, namely the inner and outer hair cells. While the inner hair cells (IHCs) are the major detectors of auditory stimuli, the outer hair cells (OHCs) enhance low level sounds by increasing the amplitude and frequency selectivity of basilar membrane vibrations, a process called “cochlear amplification” [11], [12]. From the periphery, sound information travels through the VIIIth cranial nerve to the brainstem cochlear nuclear (CN) complex, which is the primary relay station for central auditory processing [13]. This cochlear complex originates from distinct portions of the r2–r5 region, which will give rise to the anteroventral (AVCN), posteroventral (PVCN) and dorsal (DCN) cochlear nuclei, as well as to the cochlear granule cell population of the microneuronal shell [6]. A significant portion of the CN complex derives from a dorsal rim of neuroepithelium referred to as the auditory lip [6], [14], which is part of the lower rhombic lip and selectively expresses the transcription factor *Atoh1* (also known as *Math1*) [15], [16], [17]. Variously processed sound-related signals, ultimately leading to qualitative sound perception, travel from the cochlear nuclei through the lateral lemniscus (LL) complex to the inferior colliculus (IC; midbrain) and medial geniculate nucleus (MG) of the thalamus, which in turn relays auditory information to the auditory cortex. On the other hand, temporal and spatial sound localization are relayed by the cochlear nuclei through a parallel pathway in the ventral brainstem, before reaching high level brain structures [18]. This pathway includes the superior olivary complex (SOC), which is mostly derived from r5 and is partly composed of the corresponding *Atoh1^+^* lineage [3], [16], [19].

Proper hearing function is also controlled by centrifugal (efferent) motor connections, which modulate the incoming afferent sensory auditory information. The major component is represented by the olivocochlear neurons (OC), a subpopulation of the inner ear efferent (IEE) neurons, which are born in ventral r4, cross the midline during early development and segregate from their vestibular counterpart around embryonic day 14.5 of gestation (E14.5) in mice [20], [21]. While lateral OC (LOC) motor neurons innervate afferent sensory neurons in contact with the IHCs, modulate cochlear nerve excitability and protect the cochlea from neuronal damage in acute acoustic injury [21], [22], medial OC (MOC) motor neurons are innervated by reflex neurons of the PVCN [23], [24] and regulate the vibrating OHCs in the cochlea, modulating in this way the “cochlear amplification” process [25], [26], [27]. This is known as the MOC reflex. Cochlear efferent motor neurons also play a role in the normal maturation of afferent responses, particularly during the early postnatal period [21], [28]. Another feedback reflex, the middle-ear muscle reflex (MEM), is closed by facial and trigeminal branchiomotor neurons (FBM, TBM) that activate the stapedius and tensor tympani muscles respectively, thus tensing the chain of tympanic ossicles and reducing the amplitude of sound transmission through the middle ear [29], [30]. Thus, the MOC and MEM reflexes represent two parallel sound-evoked feedback mechanisms acting on the auditory periphery to modulate incoming acoustic stimuli [29], [31], [32].

Little is known about the molecular determinants involved in the assembly of rhombomere-derived auditory sub-circuits. The *Hox* genes, a large family of homeobox-containing genes, display rhombomere-restricted expression patterns and provide early patterning information to progenitors and their neuronal derivatives [33], [34]. In turn, the expression of several *Hox* genes is maintained through later stages of circuit formation in distinct, rhombomere-derived neuronal subpopulations contributing to portions of hindbrain sensory and motor nuclei [4], [7], [35], [36]. In the developing hindbrain, *Hoxb1* selectively expressed in r4 is required to pattern r4-derived ventral efferent neurons, such as IEE and FBM, and to maintain normal levels of *Hoxb2* in r4 [37], [38]. *Hoxb2* and *Hoxa2*, unlike *Hoxb1*, are expressed in r2 to r5 auditory derivatives. Moreover, the expression of *Hoxb2* and *Hoxa2* is maintained in the ventral CN, ventral nucleus of LL (VLL), and SOC nuclei throughout prenatal and postnatal developmental stages [36]. Thus, *Hox* genes are prime candidates to be involved in the assembly of sensorimotor functional circuits in the developing hindbrain.

In this study, we find that rostral rhombomeres and their associated *Hox* genes are required in establishing and maintaining two major functional circuits in the central auditory system, which have different rhombomeric origins. Firstly, we carried out a detailed fate map of r4 derivatives by generating a novel, highly restricted, r4-specific *Cre*-recombinase driver. We show that cells originating from r4 significantly contribute to the VLL, an important relay station in the sound perception pathway. Furthermore, we found that r4 largely supplies cells to the PVCN and DCN, a finding largely in agreement with previous work [6], [16], but not to the granule cells of the microneuronal shell. Secondly, in *Hoxb1* and *Hoxb2* mutants, the VLL, PVCN, and MOC motor neurons are selectively affected, though with different severities, leading ultimately to elevated auditory thresholds in adult mutant mice. Thirdly, *Hoxb1* negatively modulates *Hoxa2* during r4 patterning, whereas *Hoxa2* is mainly required in r2/r3 AVCN-derived development. Moreover, early conditional *Hoxa2* inactivation in rhombic-lip derivatives selectively perturbs the AVCN axonal pathfinding to the medial nucleus of the trapezoid body (MNTB), resulting in decreased contralateral and increased ipsilateral targeting of MNTB due to the down-regulation of *Rig1*, the main axon guidance receptor for midline crossing. Altogether, this study provides, for the first time, genetic and functional evidence for a *Hox* gene network in the establishment and maintenance of proper auditory rhombomere-specific circuitry during hindbrain development.

## Results

### Mapping of rhombomere 4-specific contribution to the auditory system

**Figure 1:**
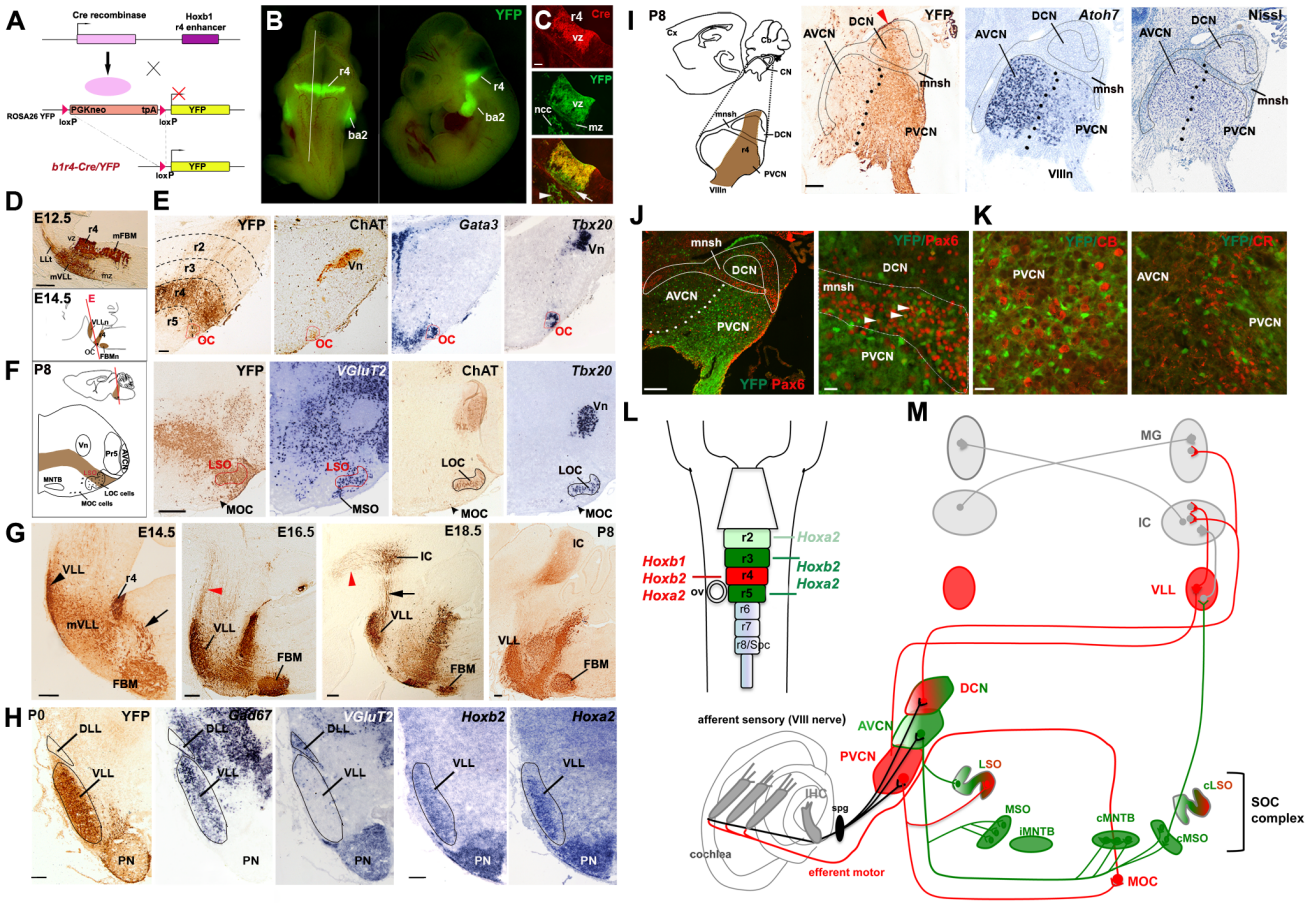
Rhombomere 4 neuronal derivatives contribute to nuclei involved in auditory perception. (A) Strategy for the *Cre-loxP* recombinase r4-fate map. Upon *Cre*-mediated recombination, the *loxP* sites surrounding the *PGKneo* cassette of the ROSA26 YY reporter line are excised and YFP is expressed exclusively in r4 and r4 derivatives. (B) Dorsal and lateral views of a E10.5 *b1r4-Cre/YFP* embryo show restricted expression of YFP in r4 and neural crest-derived cells (ncc) in the second branchial arch (ba2). The white line delineates the level and plane of sagittal section of panels in (C). (C) r4-restricted immunostaining of *Cre-recombinase* in progenitors of the ventricular zone (vz). YFP^+^/Cre^−^ post-mitotic cells at the marginal zone (mz) originate from YFP^+^/*Cre* ^+^ progenitors. (D) Sagittal sections of an E12.5 *b1r4-Cre/YFP* embryo immunostained with a GFP antibody reveal the r4 domain, the caudal migration of facial branchiomotor neurons (mFBM), the ventricular to pial migration of presumptive lateral lemniscus cells (mVLL) and the lateral lemniscus tract (LLt) projecting rostrally. Below, a schematic of an E14.5 sagittal section indicating the position of the various nuclei. The red line delineates the plane of section of panels (E). (E) The olivocochlear (OC) neurons (delimitated by a red contour) express choline acetyltransferase (ChAT), *Gata3* and *Tbx20*. (F) Schematic coronal section of a P8 brain illustrating the positions of the various nuclei. The lateral superior olive (LSO) but not the medial superior olive (MSO) nucleus, has an r4 origin, as indicated by YFP and *VGlut2* staining. ChAT and *Tbx20* are expressed in lateral (LOC) and medial OC (MOC) neurons within the LSO and ventral to the LSO, respectively. (G) Sagittal sections at different ages indicating the YFP^+^ r4-derivatives: the ventral lateral lemniscus (VLL) (rostrally) and FBM (caudally) nuclei. VLL and cochlear neurons project rostrally to the inferior colliculus (IC) and some fibers continue to the thalamus (arrowheads). (H) Coronal sections of P0 pups indicate high contribution of r4/YFP^+^ cells to the VLL, positive for *Gad67*, but not to the dorsal LL (DLL), which is *VGlut2^+^*. *Hoxb2* and *Hoxa2* are expressed in the VLL and pontine nuclei (PN). A few dispersed YFP^+^ cells are identified in the PN. (I) Schematic of a P8 brain sagittal section showing the position of the cochlear nuclear complex (CN) and its subdivision into anteroventral (AVCN), posteroventral (PVCN) and dorsal (DCN) nuclei. Adjacent sagittal sections illustrate a high contribution of YFP^+^ cells to the PVCN and DCN. The arrowhead indicates the origin of r4-migrating cells. Dots delineate the presumptive boundary between AVCN and PVCN. Only a few YFP-positive cells label the AVCN, which is highly positive for *Atoh7*. The small intensely basofilic granule cells confined to the microneuronal shell (mnsh and outlined) and identified by Nissl and Pax6 expression (J) are not positive for YFP, indicating that cochlear granule cells do not have an r4-origin. (K) Similarly, YFP^+^ cells do not co-localize with calbindin- (CB) and calretinin- (CR) expressing cells in the PVCN region. (L) Schematic of the hindbrain in which rhombomeres 2 to 5 and their respective *Hox* genes are color-coded. The same code refers to (M). (M) Overview of the two main central auditory pathways, the MOC reflex and their rhombomeric origin. While r4-derivatives (in red) contribute mainly to the ascending sound perception pathway, which runs from the CN through the VLL to the IC, r2-, r3- and r5-derivatives (in green) contribute mainly (but not exclusively) to the pathway running through the superior olivary complex (SOC), which function in the localization of the temporal and spatial origins of sounds. The MOC reflex comprising of sensory PVCN interneurons and motor efferent OC neurons is also an r4-derivative. Vn, trigeminal motor nucleus; MNTB, medial nucleus of trapezoid body; Pr5 principal sensory trigeminal nucleus. Scale bars, 100 µm (C), 200 µm (D–J left), 20 µm (J right, K). See also Figures S1 and S2.

Taking advantage of specific r2- and r3/5-*Cre*-expressing mouse lines [39], [40], previous studies have genetically mapped the contribution of the r2–r5 rhombic lip to distinct portions of the CN and SOC complexes [6], [16], [19]. However, previous attempts to generate r4-specific lines invariably resulted in additional *Cre* expression caudal to r4 [7], [16], [41]. In this study, we generated a novel mouse transgenic line, named *b1r4-Cre*, which allowed us to exclusively map r4 and its derivatives throughout the mature brainstem (Figure 1A and Figure S1). To this purpose, we used a well characterized enhancer from the *Hoxb1* locus [42] to drive the *Cre-recombinase* gene exclusively in r4 (Figure S1A). In *b1r4-Cre* transgenic animals, onset of *Cre* expression in presumptive r4 occurs first in a mosaic fashion (Figure S1C), but from E9.0 onwards, *Cre* is expressed throughout r4 as shown in Figure S1D. To permanently label the polyclonal population of cells derived from r4, the *b1r4-Cre* transgenic line was mated to the *ROSA26-YFP* reporter mouse [43] and progenies positive for both alleles (herein called *b1r4-Cre/YFP*) were analyzed (Figure 1A). Accordingly, at E10.5 activation of YFP was observed exclusively in r4 and its cellular progeny, as shown by double staining of *Cre* and YFP (Figure 1B, 1C). No ectopic expression of *Cre* was detected at later stages (data not shown).

We next analyzed the *b1r4-Cre/YFP* mouse line at different embryonic stages and early postnatal ages, using an anti-GFP antibody that cross-reacts with the YFP protein and labels proliferative and migrating/differentiating cells and their axonal projections. The r4 radial histogenetic territory itself is massively labeled and becomes morphogenetically deformed into a wedge-shaped, dorsally compressed configuration (Figure 1D, 1G). The first cohort of cells migrating out of r4 consists of the well-described caudomedial stream of tangentially migrating FBM neurons, which first move caudolaterally into r6 and then reach their definitive ventrolateral subpial position by radial migration [44], [45], [46] (Figure 1D, 1G). Another r4-derived efferent population is represented by OC neurons [20], [21], a subpopulation of IEE neurons. At E14.5 they form a compact superficial group of cells at the r4/r5 margin and selectively express the cholinergic marker ChAT [21] and the transcription factors *Gata3* and *Tbx20* [47], [48] (Figure 1E). At P8 the OC neurons split into lateral (LOC) and medial (MOC) components [49], which express ChAT and *Tbx20* and become located within the lateral superior olive (LSO) and in the medioventral portion of the SOC as scattered cells, respectively (Figure 1F). A part of the LSO, expressing the glutamatergic marker *VGlut2* [16], is included within the r4 domain (Figure 1F).

Another sizeable stream of labeled r4-derived cells, which was not previously described, migrates to the basal longitudinal zone and then moves rostrally along the growing lateral lemniscus tract, which courses obliquely through the rostral hindbrain (Figure 1D, 1G). These cells will contribute to the majority of the VLL from E14.5 onwards (Figure 1G). At E16.5, labeled ascending lateral lemniscus fibers, originating from the ipsilateral r4-derived VLL neurons and also from the r4-derived projection neurons of the contralateral CN [50], reach the IC. At E18.5, lateral lemniscus fibers also extend along the brachium of the IC into the medial geniculate nucleus of the thalamus, and collaterals can be distinctly seen in the superior colliculus intermediate layers at P8 (Figure 1G, and data not shown). Transverse sections at P0 clearly show a high density of YFP^+^ cells in the VLL but not in the dorsal nucleus of LL (DLL), which expresses the glutamatergic marker *VGlut2* and originates from the *Atoh1^+^* lineage [19], [51]. The majority of VLL neurons express the inhibitory GABAergic/glycinergic marker *Gad67*, a particular feature of this auditory structure, as previously described [52], and *Hoxb2* and *Hoxa2* [36]. In summary, our fate map study shows for the first time that r4 massively contributes to the VLL within the LL complex.

Next, we mapped precisely r4 contribution to the plurisegmental CN complex (Figure 1I). Previously, the contribution of r4 was indirectly inferred from the mapping of r3 and r5 derivatives, or from the mapping of the territory posterior to r3 [6], [16]. These studies indicated that the AVCN is derived from r2 and r3, the PVCN from r3 and r4, and the DCN from r4 and r5 (summarized in Figure 1L, 1M). A significant proportion of these nuclei were strongly affected in *Atoh1* conditional and null mutants, supporting their origin from the *Atoh1^+^* auditory lip [16], [17].

Our results are largely in agreement with previous data and further extend them. First, we show that at E10.5 the YFP^+^ domain includes *Atoh1*-expressing cells in the rhombic lip region of dorsal r4 (Figure S2A). However, at E14.5 when *Atoh1^+^* cells migrating from r2 to r5 rhombic lip invade the presumptive cochlear complex [17], only a few r4-derived YFP^+^ cells overlap with *Atoh1* expression domain, whereas no co-localization of YFP with the granule cell marker *Barlh1* [6] is found in this region (Figure S2B). Secondly, we show that YFP^+^ cells contribute to an intermediate sector across the CN complex, which crosses dorsoventrally the magnocellular core portion of the DCN and then gives rise to the majority of the PVCN (Figure 1I and Figure S2C–S2E). Small portions of the DCN remain unlabeled, suggesting additional contribution from r5, as previously reported [6], [16], but also a likely contribution from r3 to the region of DCN anterior to the YFP^+^ domain. Thirdly, we found that only a small number of YFP^+^ cells are distributed in the AVCN at P8, which, unlike the PVCN, expresses high levels of *Atoh7* (also known as *Math5*) [53] (Figure 1I). Finally, we started to characterize the cellular identity of YFP^+^ cells and found that at P8, r4-derived cells fail to co-express Pax6 (Figure 1J), a marker for the microneuronal granule cell population [15], [54]. Moreover, YFP signal is absent in calbindin- and calretinin-expressing neurons (Figure 1K), corresponding to octopus and globular-bushy cells, respectively [55], [56]. These data indicate that subpopulations of glutamatergic neurons, which normally derive from the *Atoh1^+^* neuroepithelial domain [15], do not originate from r4. A full characterization of YFP^+^ cells in embryonic and adult stages will be reported elsewhere (M.D., L.P. and M.S., in preparation).

In summary, we show that r4 largely contributes to the motor cochlear efferent neurons, to the relay VLL neurons, and within the cochlear nucleus, to the majority of the PVCN, and part of the DCN. Thus, while r4 seems to be required for the structures involved in sound transmission, amplification and protection (i.e. PVCN, VLL, and OC), r2, r3 and r5 are likely contributing to components of the sound localization pathway that runs through the SOC and trapezoid body complex before reaching higher-order auditory structures (Figure 1L, 1M).

Overall, our r4 fate mapping, combined with previously published r2, r3 and r5 maps [6], [16] and patterns of early and late *Hoxa2* and *Hoxb2* expression profiles [36], strongly suggest rhombomere-specific and *Hox*-dependent assembly of the two main central auditory sub-circuits (Figure 1L, 1M).

### Regulation of *Hoxb1*, *Hoxb2*, and *Hoxa2* in sensory r4 of *Hox* mutant mice

Previous work dissected the genetic and regulatory network involved in establishing and maintaining the identity of r4 progenitors [34]. *Hoxb1* plays a key role in patterning ventral r4 progenitors, partly through transcriptional regulation of *Hoxb2* and *Hoxa2* [37], [38], [57]. Maintenance of *Hoxb1* expression in ventral r4 requires both Hoxb1 itself and Hoxb2 through auto- and cross-regulatory interactions, respectively [37], [58], [59], [60]; however, it is not known whether a similar mechanism is acting in dorsal/sensory r4. Unlike *Hoxb1*, *Hoxb2* and *Hoxa2* expression is maintained in differentiated r4-derivatives, such as the VLL and VCN ([36] and our study). Thus, *Hoxb1* may pattern sensory r4-derivatives by regulating dorso-ventral *Hoxb2/Hoxa2* expression in r4 progenitor cells, hence controlling post-mitotic specification during the development of the sensory system. To test this hypothesis and investigate the roles of *Hoxb1*, *Hoxb2*, and *Hoxa2* in the development of auditory pathways, we analyzed the effects of their functional inactivation.

**Figure 2:**
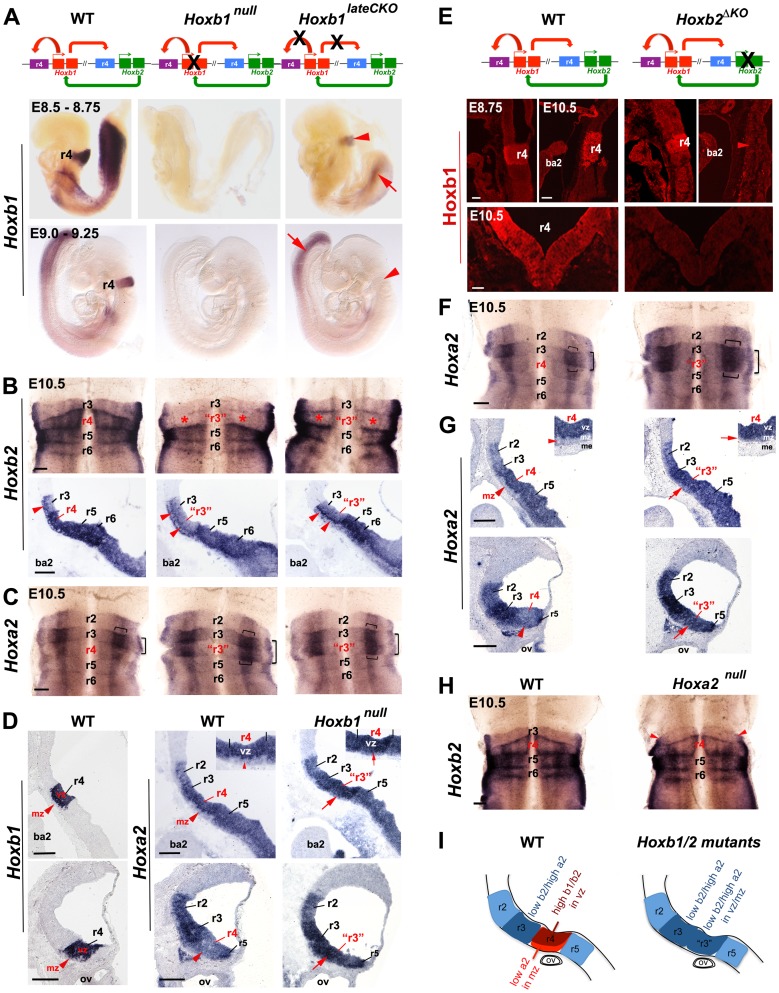
Regulatory interactions between *Hoxb1*, *Hoxb2*, and *Hoxa2* in r4. (A) The diagrams above the panels indicate the interactions between *Hoxb1* and *Hoxb2*. While *Hoxb1* auto-regulates its own expression in r4, it also binds to an *Hoxb2* r4 enhancer to maintain *Hoxb2* expression in r4. *Hoxb2* maintains expression of *Hoxb1* in r4. Crosses indicate loss of Hoxb1 protein in *Hoxb1^null^* embryos and loss of the auto- and cross-regulatory loops in *Hoxb1^lateCKO^* mutants. Lateral views of E8.5 to E9.25 embryos indicate that while *Hoxb1* expression is still maintained in r4 (although at lower levels) of E8.75 *Hoxb1^lateCKO^* mutants, r4 expression is completely abolished in E9.25 mutant embryos (arrowheads). Expression in the posterior region is still maintained at both ages (arrows). (B) Ventricular views of flat-mount preparations of E10.5 WT, *Hoxb1^null^* and *Hoxb1^lateCKO^* hindbrains hybridized with *Hoxb2*. Expression of *Hoxb2* is strongly decreased (but not abolished) in r4 of *Hoxb1^null^* and *Hoxb1^lateCKO^* embryos, at similar levels to r3 (asterisks). R4 acquires an expression pattern of r3, as indicated by “r3”. Down-regulation of *Hoxb2* in r4 can also be appreciated in mid-sagittal sections of mutant embryos. The line of cells expressing high levels of *Hoxb2* denotes early post-mitotic cells (arrowhead). (C) Ventricular views of flat-mount preparations of E10.5 WT, *Hoxb1^null^* and *Hoxb1^lateCKO^* hindbrains hybridized with *Hoxa2*. Expression of *Hoxa2* is increased in r4 and the characteristic *Hoxa2* expression profile of r3 is now duplicated in r4 of *Hoxb1^null^* and *Hoxb1^lateCKO^* embryos supporting an r4 to r3 change of identity. The horizontal and vertical brackets indicate higher expression domains of *Hoxa2*, respectively in a large intermediate stripe and in a thinner lateral stripe of the sensory column, the presumptive auditory column. (D) On the left, expression of *Hoxb1* indicates the position of r4 on sagittal sections. On the right, increased expression of *Hoxa2* is detected in the ventricular and mantle layers of r4 at two different dorsal levels in *Hoxb1^null^* embryos. In mutant embryos, the expression of *Hoxa2* is maintained at levels comparable to r3 in the r4 mantle zone (mz) (i.e. post mitotic neurons) with respect to WT (arrows, see also insets). (E) In *Hoxb2^ΔKO^* mutants, lack of Hoxb2 (indicated with a cross) results in failure to maintain *Hoxb1* expression in r4. Sagittal and coronal views show that Hoxb1 protein is present in r4 of E8.75 embryos, but not maintained in E10.5 *Hoxb2^ΔKO^* mutant embryos. (F) Flat-mounted hindbrain preparations hybridized with *Hoxa2* show a duplication of r3 features in r4 in E10.5 *Hoxb2^ΔKO^* mutant embryos. *Hoxa2* expression levels are increased in r4 in the absence of *Hoxb2*, similarly to *Hoxb1* mutant embryos. (G) E10.5 sagittal sections in dorsal regions indicate increased expression of *Hoxa2* in ventricular (vz) and mantle (mz) zones of r4 (arrows, see also insets) mimicking the expression profile of r3. (H) Flat-mounted hindbrain preparations of E10.5 WT and *Hoxa2^null^* embryos hybridized with *Hoxb2*. No particular expression changes can be observed in mutant embryos. Red arrowheads indicate a defect in alar r2/r3, as previously described [66]. (I) Schematics summarizing the expression of *Hox* genes in r4 of *Hoxb1* or *Hoxb2* mutants. Scale bars, 200 µm (B, C, F and H); 100 µm (E top); 50 µm (E bottom). ba2, second branchial arc; me, mesoderm. See also Figures S3, S4 and S5.

*Hoxb1* functional deletion results in an early re-patterning of the ventral r4 territory into a more anterior identity [44], [61]. To bypass the early *Hoxb1* role in r4 neuroepithelium and investigate its requirement during neurogenesis, we generated a novel *Hoxb1* conditional mutant allele, *Hoxb1^flox^* (Figures S3 and S4; see Materials and Methods) and mated it to the *b1r4-Cre* driver. Conditional *b1r4-Cre*;*Hoxb1^flox/flox^* homozygous mutant mice (hereafter referred to as *Hoxb1^lateCKO^*) are viable and fertile. Mutant embryos retain *Hoxb1* expression in r4 until E8.75–E9.0 (Figure 2A), in accordance with the timing of *Cre* expression and onset of *Cre*-mediated excision (Figure 1 and Figure S1). At E9.25, *Hoxb1* expression is no longer present in mutant r4, while the remainder of its expression is unaltered (Figure 2A).

We also obtained constitutive *Hoxb1^null^* homozygous mutants (Figures S3, S4; see Materials and Methods) that exhibit total loss of *Hoxb1* expression from its onset (Figure 2A) and lack any selectable cassette that might interfere with adjacent *Hox* genes [62], [63]. To specifically trace r4 derivatives from the control and the two distinct *Hoxb1* mutants throughout embryonic and postnatal brains, *Hoxb1^flox^* and *Hoxb1^null^* homozygous mice were mated with the *b1r4-Cre/YFP* reporter line (see Materials and Methods for mating schemes). In addition, we generated a novel, viable and fertile *Hoxb2* homozygous mutant allele (hereafter referred to as *Hoxb2^ΔKO^*), that, similarly to the *Hoxb1^null^* and unlike previously described *Hoxb2* knockout alleles [37], [58], [64], has no selectable marker left within the locus (see Materials and Methods). Finally, we made use of the previously described *Hoxa2^null^* and conditional *Hoxa2^flox^* alleles [7], [65].

Next, we assessed *Hox* cross-regulatory interactions within r4 in *Hoxb1*, *Hoxb2* and *Hoxa2* mutant alleles. Flat-mounted preparations and sagittal sections of control embryos show high *Hoxb2* expression levels in r4 to r6 and low levels in r3 (Figure 2B). In E10.5 *Hoxb1^null^* and *Hoxb1^lateCKO^* hindbrain preparations, *Hoxb2* expression is down-regulated throughout r4 to similar levels as in r3 (asterisks in Figure 2B), resulting in a duplication of r3-features (“r3” in Figure 2B). This is confirmed in mid-sagittal sections showing a decrease of *Hoxb2* expression in r4 progenitors of *Hoxb1^null^* and *Hoxb1^lateCKO^*. Expression is still maintained in early differentiating cells, similarly to r3 (arrowheads in Figure 2B). In contrast, *Hoxa2* is normally expressed at low levels in r2 and r4, and at high levels in r3, particularly in a wide intermediate stripe of the dorsal sensory column (horizontal bracket in Figure 2C) and in a thinner stripe laterally (vertical bracket in Figure 2C), the presumptive auditory column. In E10.5 *Hoxb1* mutant embryos, expression of *Hoxa2* is abnormally up-regulated in r4, predominantly in the two sensory stripes, resulting in a duplication of r3-typical features in r4 (Figure 2C). Sagittal sections at different alar plate levels of *Hoxb1^null^* mutant embryos confirm up-regulation of *Hoxa2* in the ventricular zone, and, strikingly, also in the mantle zone of r4 (arrows in Figure 2D and inset), which normally expresses low levels of *Hoxb1* and *Hoxa2* (arrowheads in Figure 2D and inset). In mutant r4, *Hoxa2* is maintained at high levels in the post-mitotic neurons, similarly as in r3. Thus, our data show that in the absence of *Hoxb1*, r4 acquires *Hox* features typical of r3, such as low levels of *Hoxb2* and high levels of *Hoxa2*, indicating a re-patterning of r4 into r3. In addition, *Hoxb1* differentially regulates *Hoxb2* and *Hoxa2* expression levels in r4, further supporting a complex regulatory interaction between *Hoxb1* and *Hoxb2/Hoxa2* in specifying r4 identity [37], [38], [57], [58], [59].

To investigate whether Hoxb2 acts similarly to Hoxb1 in r4 patterning, we assessed *Hoxb1* and *Hoxa2* expression in WT and *Hoxb2^ΔKO^* hindbrains from E8.75 to E10.5. Hoxb1 protein is lost in *Hoxb2^ΔKO^* at E10.5, though it is present at earlier embryonic stages (Figure 2E), indicating that Hoxb2 is required for maintaining Hoxb1 in r4. Similarly to *Hoxb1* mutants, *Hoxa2* expression is dramatically up-regulated throughout r4, with particular emphasis in the intermediate and lateral columns (Figure 2F), and in post mitotic neurons (arrows in Figure 2G and inset). In contrast, no changes are observed in *Hoxa2^null^* embryos (Figure 2H), apart from the r2/r3 alar defects previously described [66]. Taken together, our data show that within r4, *Hoxb2* acts mainly in maintaining high expression of *Hoxb1* and that in its absence, r4 acquires tr3-typical features, similarly to *Hoxb1* mutant hindbrains. Importantly, we found increased *Hoxa2* expression, at levels similar to r3, in post-mitotic r4 neurons of *Hoxb1* and *Hoxb2* mutants, implying that r4-derived sensory cells abnormally maintain *Hoxa2* expression throughout hindbrain development (Figure 2I).

Finally, we investigated whether *Hoxb1* and *Hoxb2* are required in the differentiation process of the IEE and OC motor neurons, which normally differentiate in ventral r4 and interact with dorsally-derived sensory structures during the development of the auditory sensorimotor circuitry. At E10.5, IEE are located in the r4 mantle zone next to the floor plate and normally express *Gata2/3*, *Isl1* and *Phox2b* [48], [59], [67] (Figure S5A, S5B). No IEE neurons are identified in E10.5 *Hoxb1^null^* and *Hoxb1^lateCKO^* embryos, as seen by the absence of *Gata2/3* expression within the pool of Phox2b^+^/Isl1^+^ motor neurons (Figure S5B). This is not due to a delay in specification, since no OC neurons, positive for ChAT and *Gata3*, located in ventral peri-olivary positions can be distinguished in E14.5 *Hoxb1* mutant embryos (Figure S5E). On the contrary, a few *Gata3^+^* and *Tbx20^+^* cells are preserved in *Hoxb2^ΔKO^* mutant embryos at E10.5 and E14.5, even if they are located in a slightly more dorsal position than in control embryos (Figure S5C, S5F). These data indicate that early IEE and late OC specification are maintained in *Hoxb2^ΔKO^* embryos despite the late absence of Hoxb1 in r4.

### *Hoxb1* is a key determinant gene in r4-derived VLL development

**Figure 3:**
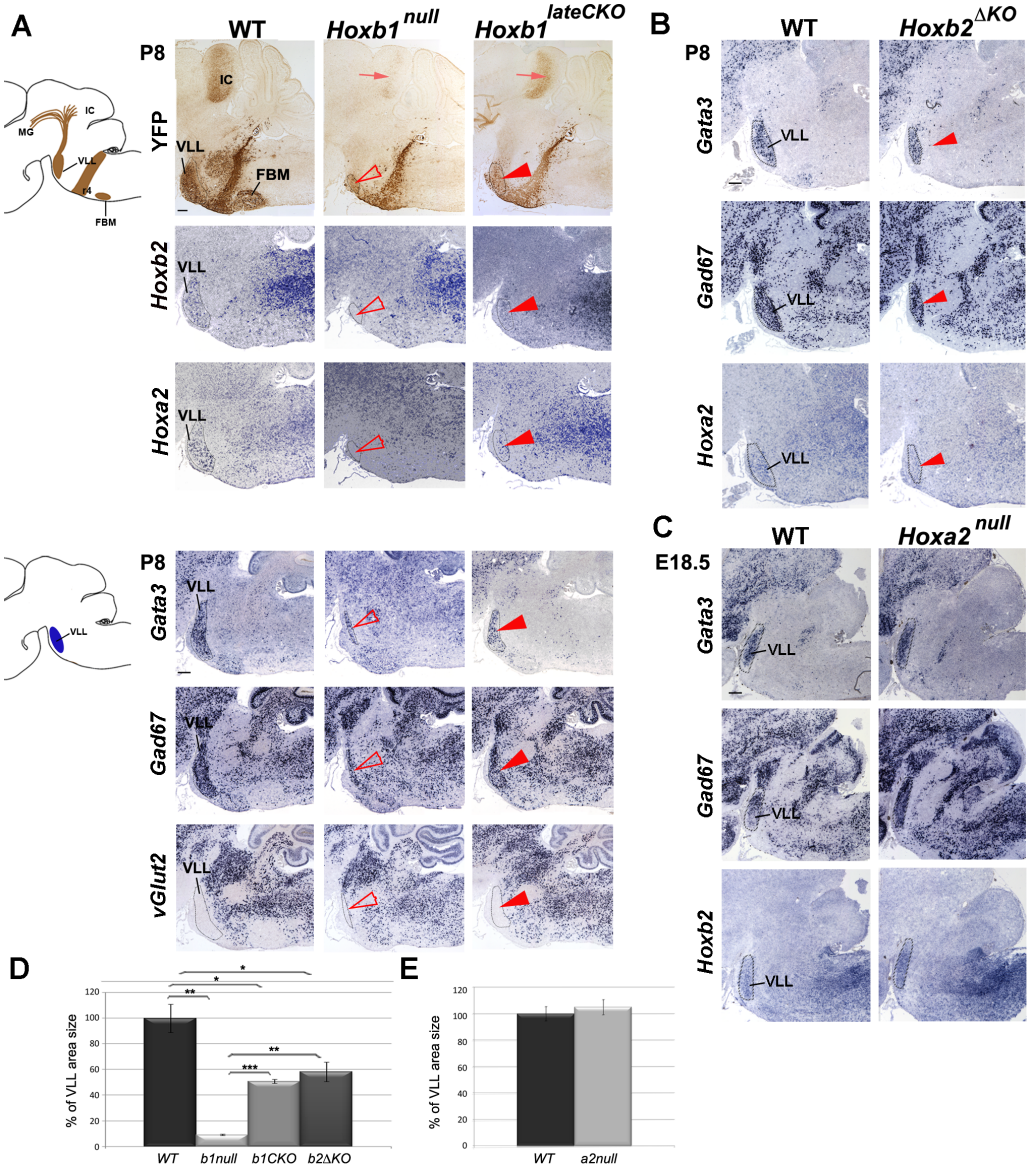
The r4-derived VLL is affected in *Hoxb1* and *Hoxb2* mutant mice. (A) Schematic view of a sagittal brain section indicating the YFP^+^ r4-derived nuclei and projections. A strong reduction of the YFP^+^ VLL nucleus (arrowhead) and projections (arrow) in *Hoxb1* mutants is observed. In constitutive mutants the reduction is much more severe than in conditional mutants, as quantified in (D). Adjacent sagittal sections show no *Hoxb2* and *Hoxa2*-expressing cells in *Hoxb1^null^* mutants, whereas cells in the reduced VLL of *Hoxb1^lateCKO^* still express *Hoxb2* and *Hoxa2*. Adjacent sections of another P8 pup confirm reduction of the VLL and indicate persistence of *Gata3-* and *Gad67-*expressing cells in both *Hoxb1* mutants. No ectopic expression of *VGlut2* is detected in the VLL region. (B) The VLL is reduced in *Hoxb2^ΔKO^* mutant pups, similarly to *Hoxb1^lateCKO^* mutants, as indicated by expression of *Gata3*, *Gad67*, *Hoxa2* and quantification in (D). (C) In contrast, *Hoxa2^null^* mutants show no significant changes in the VLL position and size quantified in (E). The apparently bigger shape is due to the slightly oblique sections in mutant compared to WT brains. (D) Histogram showing the percentage of the VLL area size in WT (set up to 100%) and in the different genotypes as indicated on the y-axis. Mutants show statistically significant differences when compared to WT, or when *Hoxb1^lateCKO^* and *Hoxb2^ΔKO^* are compared to *Hoxb1^null^* (inter-genotype comparison, ANOVA p<0.001; *Hoxb1^null^* versus WT: p = 0.001; *Hoxb1^lateCKO^* versus WT: p = 0.01; *Hoxb2^ΔKO^* versus WT: p = 0.04; *Hoxb1^lateCKO^* versus *Hoxb1^null^*: p<0.001; *Hoxb2^ΔKO^* versus *Hoxb1^null^*: p = 0.003). However, no statistically significant difference is found between *Hoxb1^lateCKO^* versus *Hoxb2^ΔKO^* (p = 0.39). (E) Histogram showing the percentage of the VLL area size in WT and *Hoxa2^null^*. No statistically significant difference is found (WT versus *Hoxa2^null^*: p = 0.56). FBM, facial branchiomotor nucleus; VLL, nucleus of lateral lemniscus; IC, inferior colliculus; MG, medial geniculate nucleus. Scale bars, 200 µm. See also Figure S6.

To investigate the requirement of *Hox* genes in the generation of sensory auditory structures, we first analyzed the size and position of the VLL with various markers on adjacent coronal sections at E18.5 and sagittal sections of P8 WT and mutant brains (Figure S6 and Figure 3). Very few cells contributing to the VLL are identified in E18.5 *Hoxb1^null^* mutant hindbrains, as shown by YFP and *Gad67* staining (Figure S6B). Only some YFP*^+^* cells with lemniscal projections, scattered rostral to the r4 wedge, are still maintained in *Hoxb1^null^* brains at P8 (Figure 3A). No detectable *Hoxb2* or *Hoxa2* expression can be found in sagittal sections at all levels, whereas reduced expression of *Gata3* and *Gad67*, which both label the GABAergic/glycinergic cellular cohort of the VLL [52], [68], is still present in the remaining VLL, which is reduced by almost 90% in area (91.9±0.02) when compared to control VLL (Figure 3A, 3D). This indicates that the early absence of Hoxb1 function prevents normal r4-derived VLL specification and/or migration, and supports a major contribution of r4 to the formation of the VLL, particularly to its GABAergic cohort, which contributes to the majority of the VLL [52]. Moreover, absence of *VGlut2* expression observed at E18.5, and confirmed at P8 (Figure S6B and Figure 3A), rules out any inhibitory to excitatory cell fate transformation within the VLL.

A less severe reduction of the VLL was observed in *Hoxb1^lateCKO^* mutants (n = 3; 49.2±0.1% in area as compared to WT; Figure 3D and legend). This was mainly supported by the maintenance of *Gata3-*, *Gad67-*, *Hoxb2*- and *Hoxa2*-expressing cells at E18.5 and P8, and by the presence, although reduced, of YFP^+^ VLL projections to the IC (Figure 3A and Figure S6B). Similarly, the VLL area is reduced by 41.7±0.3% in *Hoxb2^ΔKO^* (n = 3) when compared to WT (n = 3) brains (Figure 3D and legend), whereas no size reduction is measured in the VLL of *Hoxa2^null^* mutants (n = 3; 105±5.7%) at E18.5 before *Hoxa2^null^* perinatal death [69] (Figure 3C, 3E and legend). Together, these data indicate that *Hoxb1* is an important determinant gene in r4-derived VLL development, because r4 to r3 change of identity occurring in *Hoxb1* mutants prevent a proper VLL nucleus development. However, the persistence of some GABAergic cells in the VLL of *Hoxb1^null^* brains, suggests that other rhombomeres besides r4 might contribute to VLL formation. In contrast, *Hoxb2* or *Hoxa2* do not appear to play a predominant role in VLL specification. The reduction in VLL size in *Hoxb2* mutant brains is most probably due to the failure of *Hoxb2* maintaining *Hoxb1* expression in r4, as suggested by the lack of statistically significant differences in VLL size between *Hoxb1^lateCKO^*and *Hoxb2^ΔKO^* mutant animals (Figure 3D).

### Abnormal specification of r4-derived ventral cochlear structures in *Hox* mutants

**Figure 4:**
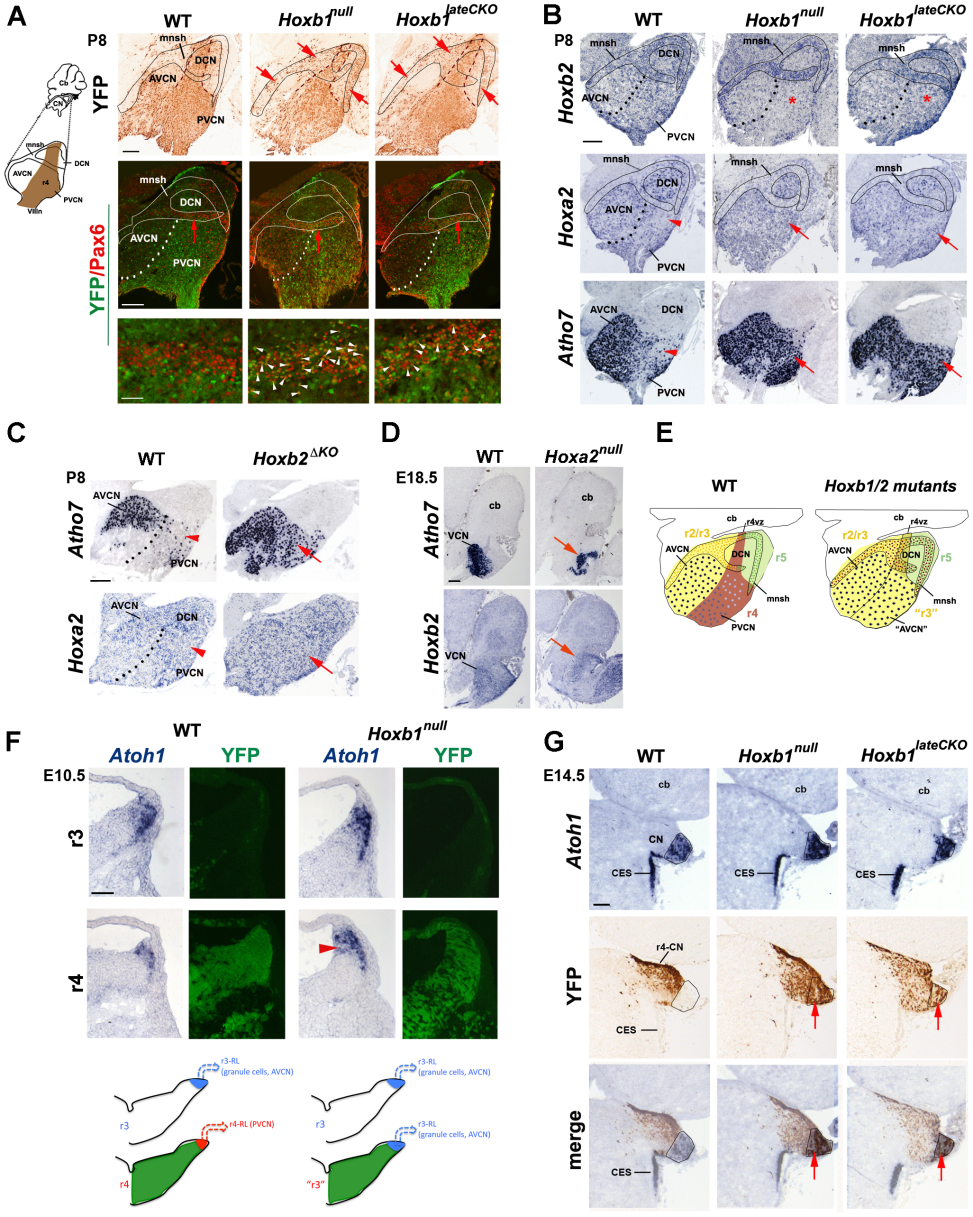
The cochlear nuclear complex is differently affected in *Hoxb1*, *Hoxb2*, and *Hoxa2* mutants. (A) Ectopic YFP^+^ r4-derived cells (arrows) are observed in the cochlear microneuronal shell (mnsh) (limited by solid line) of P8 *Hoxb1^null^* and *Hoxb1^lateCKO^* mutant sagittal sections. These cells now express Pax6 indicating that they are granule cells (white arrowheads). (B) *Hoxb2* expression is decreased in the PVCN of *Hoxb1^null^* and in *Hoxb1^lateCKO^* mutants (asterisks). On the contrary, *Hoxa2* expression is increased and *Atoh7*, normally expressed at high levels only in the AVCN, is dramatically up-regulated in the PVCN of P8 *Hoxb1* (B) and *Hoxb2^ΔKO^* mutant pups (C) (arrows). Arrowheads in WT indicate *Hoxa2* and *Atoh7* low-expressing regions. (D) Formation of the AVCN is strongly affected in E18.5 *Hoxa2^null^* brains, as seen by decreased expression of *Atoh7* and *Hoxb2* (arrows). (E) Summary schematic indicating that in the absence of Hoxb1 and Hoxb2, the PVCN (r4-derived in brown) has acquired AVCN-like features (r2/3-derived in yellow) and YFP^+^ cell (brown) contribute to the shell. (F) The dorsal-most regions of WT and *Hoxb1^null^* hindbrains at r3 and r4 levels on adjacent coronal sections hybridized with *Atoh1* and revealed by YFP epifluorescence (indicates r4 levels). *Atoh1* is expressed in progenitors and differentiating cells migrating along the lateral ridge. The *Atoh1-*expressing domain is reduced in r4 compared to r3 in WT, whereas an enlarged *Atoh1*-expressing domain (arrowhead) is identified in r4 of *Hoxb1^null^* embryos. (G) On adjacent coronal sections at E14.5, YFP^+^ (r4-derived) cells located more laterally, do not overlap with *Atoh1* ^+^ cells in the presumptive cochlear nucleus (CN), which originates from the r2–r5 auditory lip. In the absence of *Hoxb1*, YFP^+^ cells invade the *Atoh1* ^+^ domain, thus acquiring the *Atoh1* fate of adjacent rhombomeres. Cb, cerebellum; CES, caudal extramigratory stream. Scale bars, 200 µm (A up panels, B, C, D), 50 µm (A bottom panels), 100 µm (F, G). See also Figure S2.

We next investigated the involvement of *Hoxb1*, *Hoxb2* and *Hoxa2* in the development of the CN complex (Figure 4). In *Hoxb1* mutant mice, no considerable changes in the overall size of the CN were observed at P8. However, careful analysis showed that r4-derived *YFP^+^* cells massively invade the granule shell layer, normally derived from the *Atho1^+^* lineage [15], [17], [19], and ectopically expressed Pax6, as confirmed by the abnormal presence of double YFP^+^/Pax6^+^ cells in the microneuronal shell (Figure 4A). This strongly indicates that r4-derived mutant cells have now acquired a granule cell identity, which r4 does not normally contribute to (Figure 1I–1J and Figure 4A). In addition, while *Hoxb2* expression is maintained in the increased microneuronal shell of the *Hoxb1* mutants, *Hoxb2* and *Hoxa2* expression levels are slightly affected in the ventral CN (Figure 4B). Namely, *Hoxb2* is down-regulated in the PVCN (asterisks in Figure 4B), whereas *Hoxa2* expression is slightly up-regulated in PVCN regions ventral to the microneuronal shell layer (arrows in Figure 4B), in line with their respective altered expression levels observed at E10.5 (Figure 2B–2D). Notably, *Atoh7*, predominantly expressed in specific *Atoh1*-derived post-mitotic glutamatergic populations of the AVCN [15], [53], is now strongly up-regulated in the PVCN of *Hoxb1^null^* and *Hoxb1^lateCKO^* mutants, indicating that the PVCN has acquired features of the r2/3-derived AVCN [6] (summarized in Figure 4E). Next, we asked whether *Hoxb2* and *Hoxa2* are also required in the specification of VCN components. Interestingly, *Atoh7* and *Hoxa2* expressions are also increased in the PVCN of *Hoxb2^ΔKO^* (arrows in Figure 4C). In contrast, *Atoh7* and *Hoxb2* expressions are strongly reduced in the AVCN of E18.5 *Hoxa2^null^* mutants (arrows in Figure 4D), in accordance with the early patterning defect previously described in the rostral hindbrain of these mutants [66].

The dramatic increase of cells that express high levels of *Atoh7* in the PVCN and the ectopic formation of YFP^+^ granule cells observed in *Hoxb1* mutants (both are *Atoh1* ^+^ lineage derivatives), led us to hypothesize that an increase of *Atoh1* in r4 is the cause of the ectopic generation of glutamatergic neurons. Normally, the *Atoh1^+^* domain in dorsal r4 (i.e. YFP^+^) of E10.5 embryos is smaller than in adjacent rhombomeres, such as r3 (Figure 4F). In contrast, in *Hoxb1^null^* mutant embryos this domain is enlarged to a similar extent as in control r3, suggesting an up-regulation of *Atoh1* in r4, which may contribute to the acquisition of r3-like fate and hence to the ectopic generation of glutamatergic cell types (summarized in Figure 4F). This is even more pronounced at E14.5, when cells from the r2 to r5 rhombic lip contribute to the CN primordium. In WT embryos, the majority of YFP^+^ cells is identified lateral to the *Atoh1^+^* region, whereas YFP^+^ cells abnormally express *Atoh1* and clearly invade the *Atoh1^+^* domain in *Hoxb1^null^* and *Hoxb1^lateCKO^* mutants, as demonstrated by the sizeable overlap of both domains (Figure 4G). Hence, *Hoxb1* normally restricts the *Atoh1^+^* domain in r4, impinging in this way on a specific r4 dorsal fate, which is different from the *Atoh1^+^* lineage-related ones of adjacent rhombomeres. In summary, these data show that *Hoxa2* is involved in the formation of the r2/3-derived AVCN, whereas *Hoxb1* and *Hoxb2* are required in the specification of the r4-derived PVCN by imposing an r4-specific identity during auditory development.

### Axon pathfinding defects of cochlear nuclei in *Hox* mutant mice

**Figure 5:**
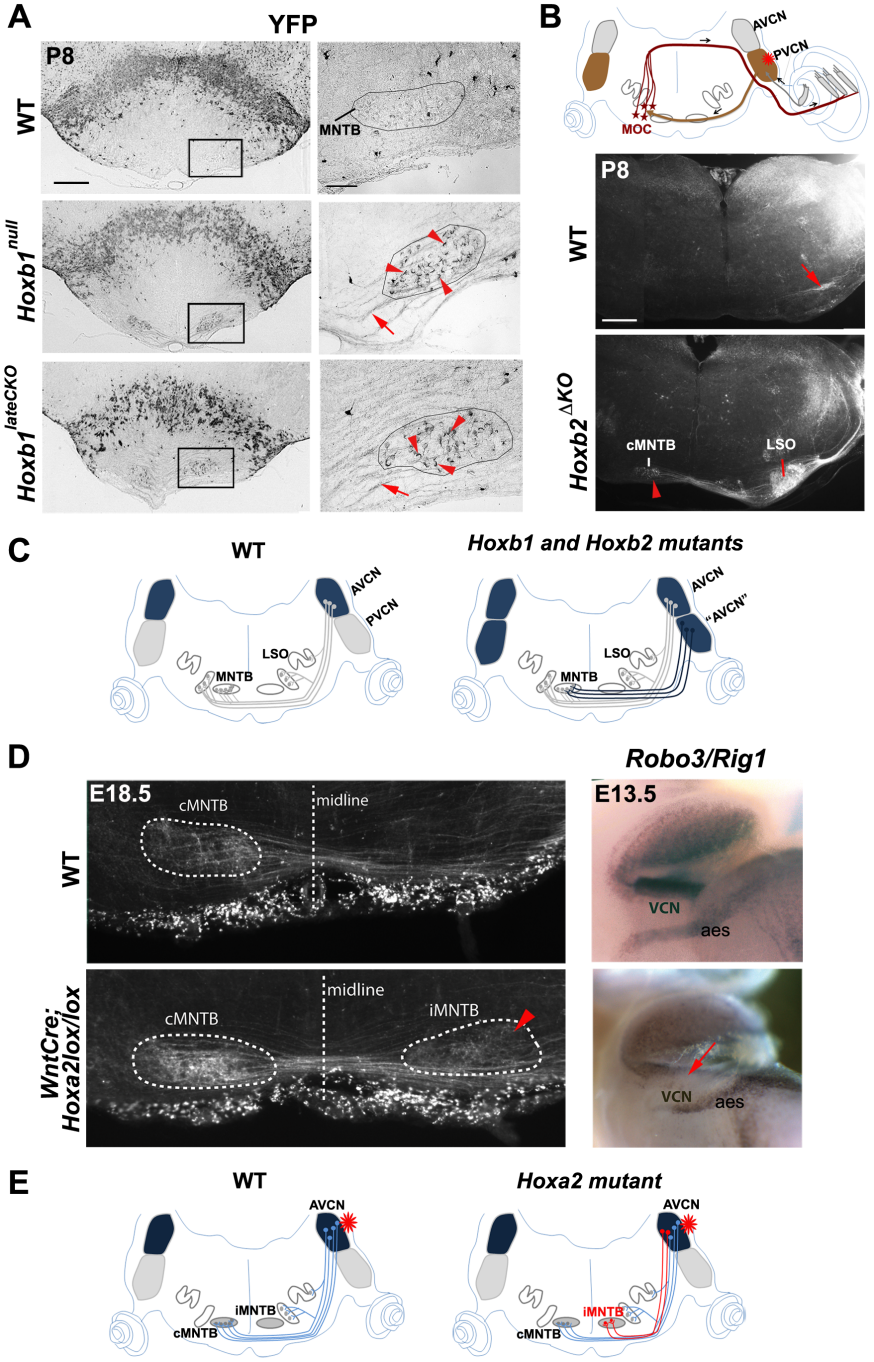
Abnormal cochlear connectivity in *Hoxb1*, *Hoxb2* and *Hoxa2* mutants. (A) Oblique P8 coronal sections show abnormal presence of YFP^+^ fibers projecting to the medial nuclei of the trapezoid body (MNTB) in *Hoxb1^null^* and *Hoxb1^lateCKO^* mutants. Right panels: enlarged views of the boxed areas to the left depicting abnormal YFP^+^ terminals (arrowheads) of labeled crossed fibers (arrows) surrounding cells of the MNTB (limited by solid line). (B) Schematic representation showing the position of the injected dextran at the level of the PVCN (asterisk). Normally, PVCN interneurons innervate contralateral MOC neurons, as indicated in the control coronal section (arrow). In *Hoxb2^ΔKO^* mutants, projections originating from the PVCN target now AVCN-specific targets, such as the contralateral MNTB (cMNTB) (arrowhead) and the lateral superior olivary (LSO) nuclei. (C) Schematics summarizing the normal connectivity pattern of cochlear AVCN neurons towards the nuclei of the superior olivary complex (SOC) complex, and the abnormal presence of YFP^+^ neurons behaving like AVCN neurons when *Hoxb1* or *Hoxb2* are inactivated. (D) A subpopulation of AVCN-labeled axons project abnormally to the ipsilateral MNTB (see arrowhead) when *Hoxa2* is conditionally inactivated in the *Wnt1^+^* (rhombic lip) domain. Expression of the Slit receptor *Rig1* (or *Robo3*) is decreased in the VCN, indicating that absence of *Rig1* affects midline crossing of AVCN projections. (E) Schematics summarizing the abnormal axonal projections of AVCN neurons in *Wnt::Cre;Hoxa2lox/lox* mice after dextran injection in the AVCN. AVCN, anterior ventral cochlear nucleus; PVCN, posterior ventral cochlear nucleus; PVCN IN, PVCN interneurons; MOC, medial olivocochlear neurons; aes, anterior extramural stream. Scale bars: 400 µm (A left panels, B), 50 µm (A right panels).

We next assessed whether deletion of specific *Hox* genes may have direct consequences on the VCN connectivity pattern at postnatal stages. In P8 *Hoxb1^null^* and *Hoxb1^lateCKO^* mutants, we identified ectopic r4-derived YFP*^+^* projections crossing the ventral midline and innervating the medial nucleus of the trapezoid body (MNTB), a normal contralateral target of r2/3 AVCN-derived fibers. These projections are never labeled by YFP in control mice, since they do not originate from the PVCN, the major source of r4-derived YFP^+^ CN projections (Figure 5A). Similarly, dextran injections in the PVCN of *Hoxb2^ΔKO^* label ectopic projections to the contralateral MNTB (cMNTB) (arrowhead in Figure 5B), whereas in control mice the very few axons projecting ventrally normally innervate contralateral MOC neurons as part of the MOC reflex (arrow in Figure 5B). This is in keeping with the finding that *Atoh7* is increased in the PVCN of *Hoxb1^null^*, *Hoxb1^lateCKO^* and *Hoxb2^ΔKO^* mutants (Figure 4B, 4C), and the notion that AVCN *Atoh7^+^* neurons normally target the MNTB [10], [53]. Thus, the molecular identity transformation of PVCN to AVCN observed in *Hoxb1* and *Hoxb2* mutant mice is further supported by abnormal connectivity to their respective targets (Figure 5C).

To directly investigate the role played by *Hoxa2* in VCN connectivity, we crossed the *Hoxa2^flox^* allele with a *Wnt1::Cre* driver [70] that allowed cell-autonomous inactivation of *Hoxa2* at early stages in rhombic lip (and neural crest) derivatives. *Wnt1::Cre*;*Hoxa2^flox/flox^* mutants die around birth due to impaired neural crest development [71], thus preventing postnatal analysis of cochlear nuclei axon connectivity and functional impact on auditory function. Nonetheless, anterograde tracing by dextran injection in the AVCN of control and *Wnt1::Cre*;*Hoxa2^flox/flox^* brains at E18.5 reveal a neuronal population aberrantly innervating the ipsilateral MNTB (iMNTB) in mutant brains (arrowhead in Figure 5D). This phenotype is reminiscent, though less prominent, of the defects observed in the *Robo3*/*Rig1* mutant mice, in which AVCN projections are prevented from crossing the midline and accumulate ipsilaterally [72]. Analysis in E13.5 control and *Hoxa2* conditional mutant mice revealed that *Rig1* expression is indeed selectively down-regulated, though not completely abolished, in the cochlear column (arrow, Figure 5D; and data not shown). Interestingly, *Rig1* expression is not affected in the anterior extramural migratory stream (Figure 5D), derived from the precerebellar *Wnt1^+^* lineage domain, where *Hoxa2* regulates the expression of the slit receptor *Robo2* [35]. Furthermore, *Rig1* expression is not affected in *Hoxb2^ΔKO^* mutants (data not shown). Thus, our data show that *Hoxa2* selectively regulates *Rig1* expression during the guidance of contralateral AVCN projections.

### Abnormal specification and innervation of olivocochlear neurons in *Hoxb1* and *Hoxb2* mutant mice

The OC neurons become subdivided into medial and lateral components (MOC, LOC). The MOC neurons support OHC maturation at early postnatal stages and regulate the prestin-induced vibration of OHCs in the cochlea [12], [21], and the LOC neurons, jointly with the MOC neurons, protect the cochlea from acoustic damage [22], [31], [32]. We next assessed whether the axonal behavior of efferent MOC neurons and synaptic MOC terminals on OHCs are affected in P8 and adult *Hox* mutant mice.

**Figure 6:**
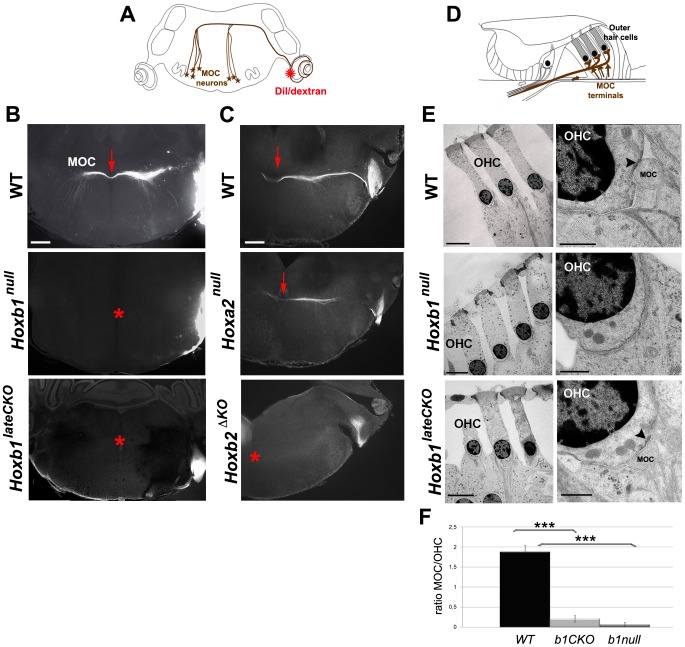
Affected connectivity of medial olivocochlear (MOC) neurons in *Hoxb1 and Hoxb2* mutant mice. (A) Schematic view of a brain coronal section indicates the insertion of a DiI or dextran crystal into the cochlea to label controlateral MOC neurons. (B) Retrogradely-labeled MOC axons normally project across the midline as a compact bundle (arrow). No MOC axons crossing the midline are retrogradely labeled in *Hoxb1^null^* and *Hoxb1^lateCKO^* brains (asterisks). (C) Similarly, MOC fibers fail to cross the midline in *Hoxb2^ΔKO^* mutant brains (asterisk), whereas no obvious defect is observed in *Hoxa2^null^* mutants (arrow). (D) Schematic view of the organ of Corti, showing terminal innervation of the OHCs by the MOC efferent neurons (arrows). (E) Transmission electron microscopy of OHCs in adults WT, *Hoxb1^null^* and *Hoxb1^lateCKO^* cochleae. In high magnification views, MOC terminals synapse on OHCs in WT and *Hoxb1^lateCKO^* cochleae, even if at much reduced ratio (F); a sub-synaptic cisterna is visualized inside the OHC (arrowheads). No MOC terminals are detected in *Hoxb1^null^* cochleae. (F) Histogram showing the ratio of the number of MOC synaptic contacts on OHC visualized in TEM experiments in controls, *Hoxb1^null^* and *Hoxb1^lateCKO^* mutants. MOC/OHCs ratio: WT (n = 3; 32 OHCs): 1.9±0.2; *Hoxb1^lateCKO^* (n = 6; 64 OHCs): 0.2±0.2; *Hoxb1^null^* (n = 4; 40 OHCs): 0.1±0.1. Inter-genotype ANOVA p<0.001; *Hoxb1^lateCKO^* versus WT: p<0.001; *Hoxb1^null^* versus WT: p<0.001. Scale bars, 400 µm (B, C), 10 µm (E, left panel of WT), 50 µm (E, left panels of *Hoxb1^null^* and *Hoxb1^lateCKO^*), 1 µm (E, right panels). See also Figures S5 and S7.

From E18.5 onwards, MOC motor axons have reached the contralateral cochlea. We thus injected DiI or dextran in *Hoxb1^null^*, *Hoxb1^lateCKO^*, *Hoxb2^ΔKO^*, or *Hoxa2^null^* mutant cochleae, and in their respective controls, to retrogradely label the fluorescent bundle of MOC efferent fibers crossing the midline (Figure 6A). No crossing axons are identified in P8 *Hoxb1^null^* and *Hoxb1^lateCKO^* mutants (Figure 6B), in keeping with the lack of OC molecular markers observed at earlier stages (Figure S5). While the MOC bundle develops normally in E18.5 *Hoxa2^null^* mutant brains, no axons cross the midline in *Hoxb2^ΔKO^* mutants (Figure 6C), albeit a few presumptive OC *Gata3^+^* cells are detected at earlier stages (Figure S5C, S5F). This suggests that, either the few *Hoxb2^ΔKO^* mutant MOC neurons fail to target the cochlea, or that our axonal tracing procedure is not sufficiently sensitive to label just a few crossing axons.

To further ascertain whether a few MOC axons may nonetheless reach the cochlea and establish synaptic contact in *Hoxb1* and *Hoxb2* mutant animals, we used transmission electron microscopy and looked for MOC terminals contacting OHCs in the organ of Corti (Figure 6D). In contrast to WT animals, in which 1 to 2 MOC terminals are normally seen in synaptic contact with individual OHCs (n = 4; 54 MOC terminals on 32 OHCs; Figure 6E, 6F), almost no MOC terminals are found in adult *Hoxb1^null^* cochleae (n = 4; 1 MOC terminal on 40 OHCs; Figure 6E, 6F). Similarly, a few residual synaptic contacts are identified in adult *Hoxb1^lateCKO^* (n = 6; 12 MOC terminals on 64 OHCs; Figure 6E, 6F) and *Hoxb2^ΔKO^* mutant cochleae (data not shown), indicating that, although in highly reduced number, some MOC neurons are able to innervate OHCs in these mutant mice (Figure 6F).

Next, we investigated whether LOC neurons were properly specified in P8 *Hoxb1* mutant mice. While ChAT and *Tbx20* label the cholinergic population of LOC neurons, *VGlut2* labels the glutamatergic population of the LSO, which is primarily derived from the *Atoh1^+^* lineage (Figure S7) [16]. No ChAT^+^ or *Tbx20^+^* cells are found in the LSO of *Hoxb1^null^* brains, whereas very few positive cells can be identified in *Hoxb1^lateCKO^* mutant brains (Figure S7B). On the contrary, *vGlut2* expression is only slightly decreased, particularly in *Hoxb1^null^* mutants, possibly because the LSO is only partially derived from r4, as previously shown [3], [16]. Thus, the populations primarily affected in *Hoxb1* and *Hoxb2* mutants are the cholinergic LOC neurons. Since the LSO largely forms within r5, this implies a previously unnoticed migration of some r4-derived cholinergic LOC cells into r5, possibly accompanying in part the migration of FBM neurons.

In summary, our molecular and cellular data confirm the absence of MOC and LOC efferent neurons in *Hoxb1^null^* mutants, but reveal the residual presence of a few MOC connections and some LOC neurons in *Hoxb1^lateCKO^* and *Hoxb2^ΔKO^* adult animals. This suggests that specification of OC neurons is primarily dependent on *Hoxb1* expression in progenitor cells and on *Hoxb2* expression in early post-mitotic neurons for their normal migratory and connectivity properties.

### Abnormal morphology of cochlear hair cells in *Hoxb1* and *Hoxb2* mutant mice

**Figure 7:**
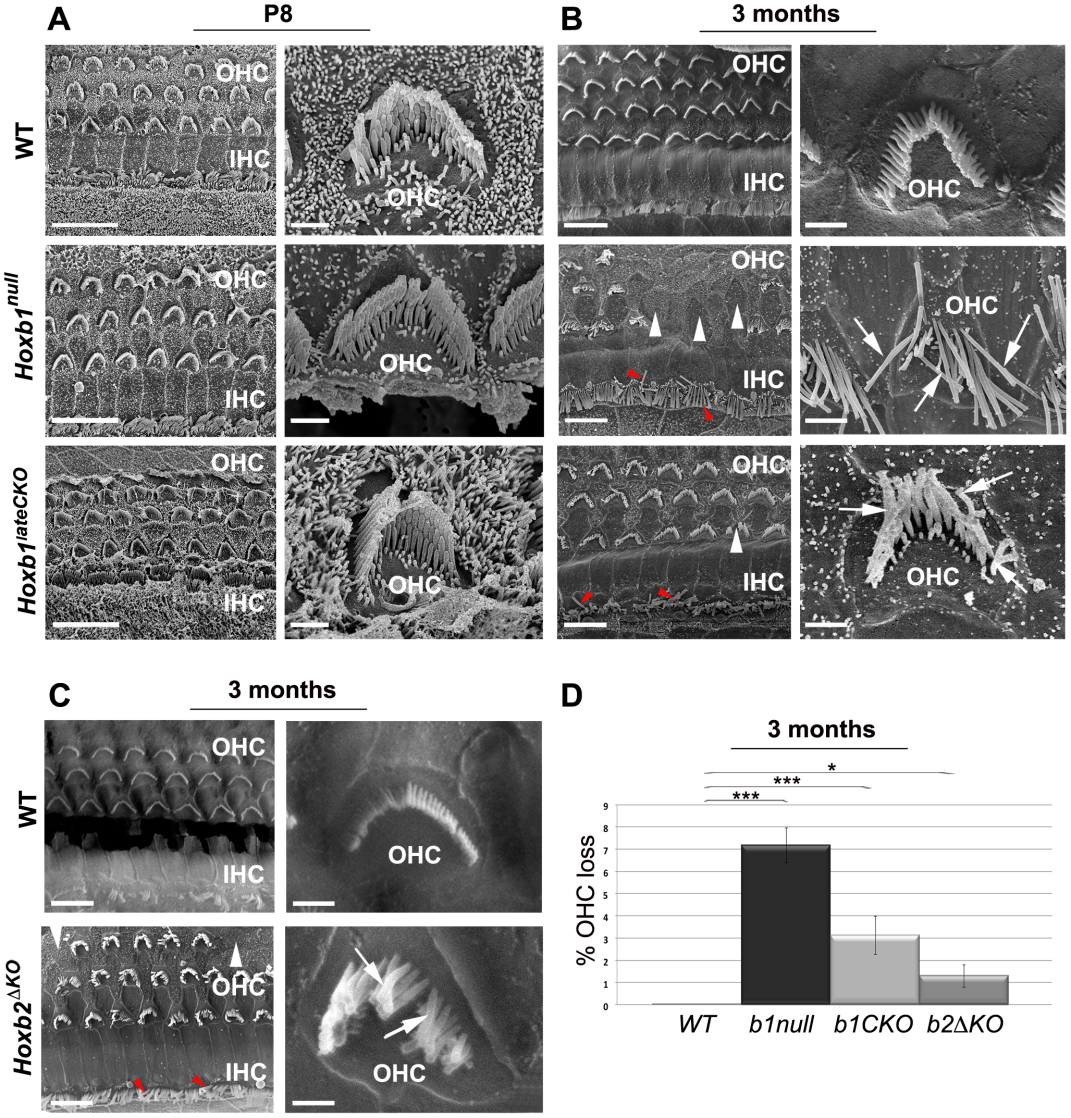
Late degeneration of OHCs in the apical turn of *Hoxb1* and *Hoxb2* mutant cochleae. (A) Scanning electron microscopy (SEM) views of the cochlea at P8: an overview of the apical turns of WT, *Hoxb1^null^* and *Hoxb1^lateCKO^* cochleae showing three orderly arrayed rows of outer hair cells (OHCs) and one row of inner hair cells (IHCs). Representative high magnification images illustrate stereocilia of hair bundles of single OHCs arranged according to their different lengths. Shape and organization of OHCs in apical regions are normal at this stage in both mutants. (B) SEM views of 3-month-old WT, *Hoxb1^null^* and *Hoxb1^lateCKO^* cochleae and representative higher magnification images of OHCs. In *Hoxb1^null^* and *Hoxb1^lateCKO^* cochleae, OHCs have lost their regular organization and fail to develop in some areas (white arrowheads). Moreover, in *Hoxb1^null^* cochleae most stereocilia have completely lost their typical V-shaped morphology and their characteristic differences in lengths (arrows). OHCs are less severely affected in *Hoxb1^lateCKO^* cochleae. IHC cilia appeared weakly disarranged (red arrowheads). (C) SEM views of 3-months-old WT and *Hoxb2^ΔKO^* mutant cochleae and higher magnifications of representative OHCs. Note that, similarly to *Hoxb1^lateCKO^* cochleae, *Hoxb2* mutants have occasional missing OHCs (white arrowheads), disarranged IHC cilia (red arrowheads) and disorganized OHC stereocilia (arrows). (D) Histogram quantifying the percentage of OHC loss in controls, *Hoxb1^null^*, *Hoxb1^lateCKO^* and *Hoxb2^ΔKO^* cochleae. While controls (n = 8) showed no OHC loss, in *Hoxb1^null^* mutants (n = 6) 7.2±0.8% of OHCs were absent, whereas 3.1±0.9% and 1.3±0.5% were lost in *Hoxb1^lateCKO^* (n = 6) and *Hoxb2^ΔKO^* (n = 3) cochleae, respectively. Inter-genotype ANOVA p<0.001; *Hoxb1^null^* versus WT: p<0.001; *Hoxb1^lateCKO^* versus WT: p<0.001; *Hoxb2^ΔKO^* versus WT: p = 0.02. Scale bars, 10 µm (A, B, C, left panels), 1 µm (A, B, C, right panels). See also Figure S8.

The failure of MOC neurons to innervate OHCs and the absence of LOC neurons innervating IHCs might affect the correct development of cochlear hair cells and/or render them more susceptible to degenerative acoustic trauma [22], [25], [28], [32], [73]. To assess hair cell morphology in *Hox* mutant cochleae, we used scanning electron microscopy on the apical and basal turns of WT, *Hoxb1^null^*, *Hoxb1^lateCKO^* and *Hoxb2^ΔKO^* cochleae. Since the medial efferent system is mature prior to the onset of hearing [21], we performed this analysis at P8, when the interactions of MOC fibers with OHCs become established, and in 3-month-old animals, when the centrifugal cochlear connections are fully functional (Figure 7 and Figure S8). In this way, we could discriminate a developmental intrinsic defect of OHCs from a defect due to the absence of MOC/OHCs interactions.

Normally, three rows of OHCs and one row of IHCs are orderly arrayed along the entire organ of Corti at both ages. High magnification images of the hair bundles of individual WT OHCs illustrate the normal three rows of stereocilia of increasing height arranged in the characteristic V-shaped morphology; the latter appears slightly wider at the basal than apical turn (Figure 7 and Figure S8; n = 8). We found no obvious differences in the shape and organization of OHCs at the apical and basal cochlear turns in *Hoxb1^null^* (n = 6) and *Hoxb1^lateCKO^* (n = 4) pups at P8 (Figure 7A and Figure S8A), indicating a normal morphological development of the cochlea in young *Hoxb1* mutant pups. However, when the architecture of the OHC area was assessed in 3-month-old animals, once the MOC/OHCs functional interactions are established and the cochlea has become fully responsive to sound, *Hoxb1^null^* adult mice show severely disorganized OHC rows with occasional loss of hair cells in the apical turn (white arrowheads in Figure 7B) (*Hoxb1^null^*, n = 6: on average 7.2/100.8 OHCs are missing, versus WT, n = 8: 0/99 OHCs missing; Figure 7D). Furthermore, close observation of individual OHCs in *Hoxb1^null^* cochleae indicates that most stereocilia lose their typical V-shaped arrangement, as well as their organized structure and characteristic differences in ciliar length (arrows in Figure 7B). On the contrary, no IHCs are lost in these mutants, even if their cilia appear weakly disarranged (red arrowheads in Figure 7B). Only slight abnormalities in ciliar organization and orientation are observed in the basal turns of *Hoxb1^null^* cochleae (arrows in Figure S8B; n = 6), indicating that the major defects occur predominantly at the cochlear apex. In contrast, in *Hoxb1^lateCKO^* and *Hoxb2^ΔKO^* cochleae, morphological defects of OHCs are less severe compared to those in *Hoxb1^null^* mutants, although a loss of OHCs is still statistically significant, with occasionally missing cells (*Hoxb1^lateCKO^*, n = 6: average of 3.1/99.4 OHCs missing; *Hoxb2^ΔKO^*, n = 3: 1.3/100.1 OHCs missing; Figure 7D) and moderate OHC and IHC ciliar malformations (Figure 7B, 7C). In any case, the major abnormalities observed in *Hoxb1* and *Hoxb2* mutants are the severe morphological defects in ciliar shape and organization, rather than the OHC loss, which was negligible, even if statistically different between the genotypes. Finally, no abnormalities are observed in basal regions of *Hoxb1^lateCKO^* and *Hoxb2^ΔKO^* cochleae (Figure S8B; and data not shown).

Taken together, we found strong late postnatal morphological defects of outer hair cells in *Hoxb1^null^* mutants, particularly in the apical region of the cochleae where low-frequency sounds are normally perceived [11]. In addition, minor defects were detected in the ciliar shape and number of hair cells in *Hoxb1^lateCKO^* and *Hoxb2^ΔKO^* mutants, indicating that abnormal development of r4-derivatives in the auditory brainstem can affect, with different severities, the long-term survival and organization of cochlear hair cells.

### Hearing loss in *Hoxb1* and *Hoxb2* mutant mice

**Figure 8:**
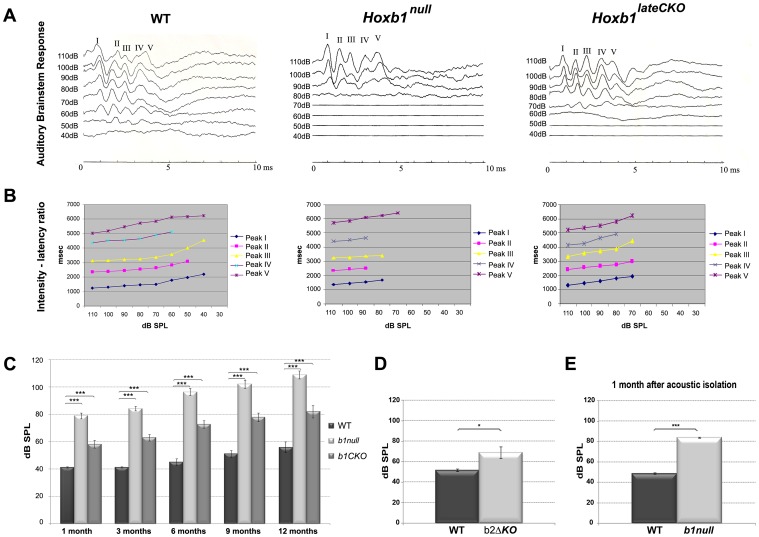
Elevated thresholds of auditory brainstem responses (ABR) in *Hoxb1* and *Hoxb2* mutant mice. (A) Representative ABR measurements of 3 month-old WT, *Hoxb1^null^* and *Hoxb1^lateCKO^* mice. Five ABR peaks are the normal sequential responses evoked by an auditory stimulus at the level of the auditory nerve and along the series of auditory nuclei of the brainstem. The threshold, the lowest intensity of sound at which the response is present, is higher in *Hoxb1* mutants than WT mice. (B) Latencies, the time intervals between the stimulus and the diverse response peaks, are normal at different intensities of sound. (C) Mean (± SE) thresholds measured in dB SPL (Sound Pressure Level) for WT, *Hoxb1^lateCKO^* and *Hoxb1^null^* mice at 1, 3, 6, 9 and 12 months of age. The differences between WT, *Hoxb1^lateCKO^* and *Hoxb1^null^* groups are statistically significant at all ages (*1 month*: WT, n = 24, 41.2±0.5; *Hoxb1^null^*, n = 25, 79.0±2.1; *Hoxb1^lateCKO^*, n = 23, 58.3±2.8; *3 months:* WT, n = 28, 41.2±0.4; *Hoxb1^null^*, n = 23, 84.3±1.7; *Hoxb1^lateCKO^*, n = 33, 63.0±2.4; *6 months:* WT, n = 22, 45.2±2.5; *Hoxb1^null^*, n = 17, 96.5±2.6; *Hoxb1^lateCKO^*, n = 25, 72.8±2.7; *9 months:* WT, n = 20, 51.2±2.5, *Hoxb1^null^*, n = 14, 102.1±3.1, *Hoxb1^lateCKO^*, n = 17, 77.9±3.1; *12 months:* WT, n = 10, 56±4, *Hoxb1^null^*, n = 12, 108.9±3.0, *Hoxb1^lateCKO^*, n = 14, 82.1±4.6; ANOVA p<0.001; post hoc t-test p<0.001; WT versus *Hoxb1^null^*; WT versus *Hoxb1^lateCKO^*: p<0.001 for all stages). A progressive increase of hearing thresholds in all groups with age is more prominent in *Hoxb1^null^* and *Hoxb1^lateCKO^* mutants than in the control mice. (D) Mean (± SE) thresholds of ABR for 3-month-old control and *Hoxb2^ΔKO^* mice. The differences between the two groups are statistically significant (WT, n = 5, 52±1.3; *Hoxb2^ΔKO^*, n = 5, 69±5.7; t-test p = 0.013); the threshold increase is similar to that of *Hoxb1^lateCKO^* mice at the same age. (E) ABR of 1 month-old WT and *Hoxb1^null^* mice put in an acoustically isolated environment from birth. Auditory thresholds are increased (WT: 48.9±0.2; *Hoxb1^null^*: 84.2±0.4; p<0,01) similarly to those of non-acoustically isolated mutants (C). dB, decibel.

The observed defects of the different components of the central auditory pathway leading to sound perception prompted us to test general auditory function in *Hoxb1* and *Hoxb2* mutant mice. We measured the auditory brainstem response (ABR) in which a series of electrical potentials evoked by auditory stimuli ranging from 110 to 40 dB SPL (Sound Pressure Level) are analyzed, determining the lowest decibel level, or threshold, at which a response peak is reproducibly present [74]. The sequence of waves of the ABR reflects the synchronous short-latency synaptic activity of many neurons in successive nuclei along the central auditory pathway. We first analyzed the ABR response in control, *Hoxb1^null^* and *Hoxb1^lateCKO^* mice from 1 to 12 months of age (n = 307). While control mice have a normal 40 dB SPL threshold, the threshold is elevated to 90 dB SPL in *Hoxb1^null^* mice representative ABRs of 3-month-old mice (Figure 8A). *Hoxb1^lateCKO^* mutant mice also show a pathologically elevated average threshold, although lower than that of *Hoxb1^null^* mice (Figure 8A, 8C). In all ages examined, the threshold values of the responding *Hoxb1* mutant mice are significantly higher than those of control mice (Figure 8C). Analyzing all data with regard to age, we observed a progressive increase of the hearing threshold for all three groups, although with different severities (doubled for *Hoxb1^null^* and 1.6 times for *Hoxb1^lateCKO^* with respect to WT), most likely due to a secondary degeneration of cochlear hair cells and/or related afferent neural structures [75]. Interestingly, our data do not show any differences in the latencies of the evoked waves (Figure 8B), suggesting that the auditory stimuli can seemingly travel normally along the successive nuclei of the central auditory pathway, once the decibel levels surpass the elevated threshold. Furthermore, we analyzed a group of *Hoxb2^ΔKO^* 3-month-old mutant mice (n = 5), together with their respective controls, and found an average threshold similar to that of *Hoxb1^lateCKO^* mice at the same age (Figure 8D).

Taken together, our data demonstrate that in the absence of Hoxb1 or Hoxb2 function, low-amplitude sounds are not perceived in young and adult mice.

### Environmental auditory stimuli are not the primary cause of auditory impairment in *Hoxb1^null^* mice

Previous studies have shown that the MOC and LOC neurons are required in protecting the cochlea against acoustic injury [22], [28], [31], [32]. Thus, the lack of OC neurons observed in *Hoxb1^null^* mutant mice may render these mice particularly hypersensitive to environmental sounds and lead to severe OHC damage and hearing defects. To directly address this issue, a group of WT and *Hoxb1^null^* pups (n = 5) were placed in an acoustically isolated environment at birth, and their hearing capacity was tested after one month of age. We found that acoustically isolated *Hoxb1^null^* mice have the same threshold shift as non-acoustically isolated mice (Figure 8E). This suggests that the environment is not the primary cause of the auditory threshold defects observed in *Hoxb1^null^* mice. However, the absence of protection from acoustic injury might determine secondarily the progressively more drastic increase of threshold observed with age in mutants compared to WT mice [76].

## Discussion

### Rhombomere 4 contribution to the auditory system

Our present r4-restricted fate map has confirmed previous studies, but also highlighted specific aspects that were not recognized before [6], [16]. In particular, we found that r4 contributes primarily to the generation and specification of auditory nuclei involved in sound transmission and amplification, as well as in the establishment of specific sensorimotor auditory circuitry during development (Figure 1M). Previous studies showed that ventral r4 is responsible for the specification of distinct subtypes of motor neurons, such as facial branchiomotor and inner ear efferent neurons [21], [44], [61], [77]; here, we show that dorsal r4 contributes to alar-plate-derived sensory components, such as the VLL, PVCN and DCN. In particular, cells migrating rostrally from r4 along the lateral lemniscus tract form the VLL nucleus, whereas dorsal sensory cells remaining within r4 contribute massively to the CN complex (PVCN and DCN), as well as to the vestibular and trigeminal sensory columns (M.D., L.P., M.S., unpublished). Interestingly, we find that r4-derived cochlear sensory neurons form jointly with basal-plate-derived motor structures two distinct auditory sensorimotor feedback sub-circuits essential for proper hearing. Some PVCN neurons together with auditory efferent MOC neurons generated within r4 support the sound-evoked MOC reflex, which terminates directly on the OHCs of the cochlea and modulates the gain of the cochlear amplifier [23], [24], [25], [26]. Interestingly, VCN neurons are also involved in the MEM reflex loop [29] through the action of the facial motor nucleus, originated in ventral r4 and strongly affected in *Hoxb1* mutant mice [44], [61] (data not shown). This implies that a single rhombomere, in this case r4, contributes to various derivatives of the auditory pathway; these are distributed across several rhombomeres via selective migrations and structurally linked into functional circuits essential for proper hearing.

We also found that the majority of r4-derivatives do not overlap with the r3/r5-derived *Atoh1^+^* lineage, which contributes to the AVCN and to the nuclei of the superior olivary complex involved in the spatial localization of sounds [16], [19]. Although further analyses are necessary to characterize the single populations derived from r4, our study suggests that r4 contributes more to inhibitory neurons (GABAergic and glycinergic) of the VLL and CN than to excitatory glutamatergic sub-populations. Firstly, we found that the VLL nucleus, which contains a majority of inhibitory *Gad67* ^+^ neurons, is mainly an r4-derivative, differently from the *VGlut2^+^* DLL, which is excitatory and originates primarily from the *Atoh1* ^+^ lineage [17], [19], [51]. Secondly, r4-derived rhombic lip cells do not contribute to the cochlear granule cell populations, nor to octopus and globular bushy cells, all of which are glutamatergic *Atoh1*-derivatives [15], [53]. Thirdly, the change of r4 to r3 identity as a result of *Hoxb1* inactivation in *Hoxb1* and *Hoxb2* mutants, leads to an increase of the excitatory populations (such as the cochlear granule cell population) and decrease of the inhibitory cell types (such as the *Gad67*- and *Gata3*-expressing populations in the VLL). Fourthly, the *Atoh7* ^+^ glutamatergic neurons, which derive from the *Atoh1*-expressing neuroepithelial regions, are massively increased in the PVCN of *Hoxb1* mutant mice. Accordingly, mutant r4/YFP^+^ neurons ectopically project to the MNTB nucleus, as normally done by AVCN cells originating from the *Atoh1^+^* lineage in r3 [16], [72]. Finally, we observed an increased dorsal r4 *Atoh1^+^* domain in *Hoxb1^null^* mutants; this may be correlated to the ectopic production of glutamatergic granule cells and *Atoh7^+^* neurons in the mutant r4-derived CN.

We therefore propose that *Hoxb1* is indirectly involved in regulating and/or modulating the ratio between inhibitory and excitatory neurons in the r4-derived auditory circuits. Since r4 is changed to a more rostral identity (r3) in absence of Hoxb1 function, the rhombomere-specific ratio between GABAergic/glycinergic and glutamatergic neuronal fates might be consequently altered. Previous studies have shown that *Ptf1a* determines GABAergic over glutamatergic neuronal cell fate in the spinal cord and cerebellum [78], [79], [80], and is required for inhibitory GABAergic and glycinergic fate in the cochlear nucleus [15]; hence, it is plausible to speculate that *Ptf1a* expressed in the r4 ventricular zone may act downstream of, or together with Hoxb1 in the determination of r4-specific inhibitory features; altered *Ptf1a* expression may be responsible for the loss of GABAergic neurons in the VLL and/or for the ectopic expression of *Atoh1*, production of glutamatergic granule cells and *Atoh7* ^+^ neurons in the r4-derived CN.

However, some *Atoh1^+^* cells do derive normally from r4, even if the corresponding progenitor domain is reduced compared to that of other rhombomeres, and might thus also partially contribute to the glutamatergic lineage. Intersectional long-term fate mapping between r4 and neuronal subtype-specific mouse lines together with careful characterization of individual sub-populations will be required to fully elucidate this aspect.

### Distinct regulation of *Hoxb2* and *Hoxa2* expression by *Hoxb1* during patterning of sensory r4-derived neuronal structures

Previous studies reported that Hoxb1 together with Hoxb2 are crucial in impinging on an r4 identity during rhombomere patterning, and that ventral r4 is changed into a more anterior identity in the absence of Hoxb1, based on ectopic expression of markers and abnormal behavior of FBM neurons [34], [37], [38], [44], [61]. Here, we show that a similar regulation occurs also in dorsal r4 during the specification of auditory sensory derivatives. We found a re-patterning of r4 into r3 identity in *Hoxb1* mutants, in which *Hoxb1* is either constitutive (*Hoxb1^null^*) or conditionally (*Hoxb1^lateCKO^*) eliminated in r4, and in *Hoxb2^ΔKO^* mice, in which *Hoxb1* expression fails to be maintained in r4. In the absence of *Hoxb1*, *Hoxb2* expression is reduced and *Hoxa2* expression is increased in r4 at levels similar to those in r3, leading ultimately to the loss of specific r4-derived auditory nuclei (VLL and PVCN) and the ectopic formation of r3-like derived structures (AVCN and cochlear granule cells).

We thus conclude that within this sensory system Hoxb1 and Hoxb2 are critically required during the specification of r4-derived structures, and that in their absence, r4 alar and rhombic lip derivatives largely acquire r3-like features. This is based on our data and is consistent with previous intersectional fate mapping with r2 and r3/r5-specific enhancers together with the selective inactivation of *Atoh1* in r3 and r5 [6], [16].

In addition, we observed that *Hoxb2^ΔKO^* and *Hoxb1^lateCKO^* reproduce a similar phenotype. Indeed, although early *Hoxb1* expression is able to partially specify r4 identity in *Hoxb1^lateCKO^* and *Hoxb2^ΔKO^* mutants, failure to maintain *Hoxb1* at later stages inhibits further development of r4-derived structures, leading ultimately to a milder phenotype when compared to constitutive *Hoxb1* null mutants. We observed slight differences in the phenotypic severity of these two mutant lines, which might essentially be due to the differences in timing of *Hoxb1* inactivation in r4.

Importantly, our data show that in r4 *Hoxb2*, besides being involved in maintaining high levels of *Hoxb1* in progenitor cells (our study and [37], [58]), also plays a key role in relaying r4-dependent regional fate to post-mitotic cells. In support of this, we show that *Hoxb2* is expressed in both r4-derived progenitors and post-mitotic neurons, and that its expression is maintained in r4-derived structures, such as the VLL, VCN and OC.

A different situation applies for *Hoxa2*, which plays crucial roles in cell migration and axonal connectivity during hindbrain development, and whose expression is also maintained in the auditory sensory nuclei [36], [66]. *Hoxb1* expression is not affected in *Hoxa2^null^* mutants, as previously reported [66] and, accordingly, no defects were observed in VLL and MOC development, two r4-derived structures. However, we found that *Hoxa2* expression in r4 is increased in *Hoxb1* and *Hoxb2^ΔKO^* embryos, both in progenitors and, importantly, in post-mitotic cells. This ectopic expression is maintained in the postnatal PVCN of these mutants together with higher expression of *Atoh7*, ultimately leading to a change of PVCN to AVCN identity, as confirmed by a corresponding abnormal connectivity pattern. Thus, we propose that Hoxb1 negatively modulates *Hoxa2* expression levels in r4. In the absence of Hoxb1, ectopic up-regulation of *Hoxa2* in PVCN changes r4-specific neuronal properties and drives cochlear neurons to innervate an inappropriate target, the MNTB cells, normally innervated by r3-derived AVCN axons [72].

Taken together, we show that while *Hoxb1* and *Hoxb2* establish and maintain the regional identity of r4 progenitors (*Hoxb1* and *Hoxb2*) and possibly of their derivatives (*Hoxb2*), *Hoxa2* is normally expressed at low levels in differentiating cells of r4 and thus plays only a minor role in patterning r4-derived sensory structures.

### *Hoxa2* regulates axon guidance receptor *Rig1* expression in the auditory lip

In this study, we found that in AVCN, *Hoxa2* controls the expression of the Slit receptor *Rig1/Robo3*, known to regulate crossing of the midline by commissural axons in the hindbrain [72]. In *Wnt1::Cre;Hoxa2^flox/flox^* mutants, in which *Hoxa2* is conditionally inactivated solely in rhombic lip and neural crest cells, the CN complex is properly formed but many axonal projections from the AVCN fail to reach the contralateral MNTB, indicating that *Rig1* is directly involved in these axonal defects.

Concerning possible ways by which Hoxa2 may regulate *Rig1* expression, it is noteworthy to mention that *Rig1* expression is not affected in the anterior extramural migratory stream derived from the posterior precerebellar *Wnt1^+^* domain, where *Hoxa2*, instead, directly regulates the expression of another Slit receptor, *Robo2* [35]. Thus, *Hoxa2*-mediated regulation of *Rig1* is mostly evident in the most rostral domain of Hoxa2 activity (i.e. in the r2–r5-derived auditory lip), whereas in the r6–8-derived precerebellar lip, the role of Hoxa2 may be functionally compensated by other *Hox* factors of the paralogue groups 3–5. Alternatively, *Hoxa2*-dependent regulation of *Rig1* might require a specific co-factor only present in r2–r5 rhombic lip derivatives. Finally, while Hoxa2 is required for the expression of *Rig1* throughout the r2–r5 auditory lip column, *Rig1* is normally expressed in *Hoxb2^ΔKO^* mutants (data not shown), strongly suggesting that *Hoxa2* expression in *Hoxb2* (and, likely, *Hoxb1*) knockout cells is sufficient to support normal *Rig1* transcriptional regulation and thus, drive the contralateral ectopic projections of ‘PVCN’ mutant neurons. However, based on the available Rig1 functional data [72], we predict that the Rig1 function by itself is not sufficient to switch PVCN-to-AVCN target specificity (i.e. to target the MNTB), holding that *Rig1* expression only confers the ability of axons to cross the midline. Thus, it is unlikely that the *Hoxa2*-mediated regulation of *Rig1* alone could explain the target connectivity switch observed in *Hoxb1* and *Hoxb2* knockouts. Hoxa2 likely controls a larger downstream transcriptional program to provide r2/3 AVCN neurons with their proper regional identity and connectivity.

### Assembly of a sensorimotor auditory sub-circuit by *Hoxb1* and *Hoxb2*

We show that *Hoxb1* and *Hoxb2* mutants have increased auditory thresholds leading to severe hearing impairments. This phenotype is often associated with affected CN function [16] and/or alterations in the cochlear amplification mechanism executed by the OHCs [11], [12], [81]. Accordingly, we found defects in the CN complex and additional strong morphological damage of the OHCs. We exclude a direct role of *Hoxb1* and *Hoxb2* on hair cell development, since they are not expressed in presumptive hair cells [41], [82]. We also exclude that a defect in satellite glial cells, surrounding the spiral ganglion neurons and originating from r4-derived neural crest (Figure S9), can affect OHC development and/or contribute to the altered auditory threshold. Even if we observed a decrease of double YFP^+^/Sox10^+^ cells in *Hoxb1^null^* cochleae, spiral ganglion neurons seem to differentiate properly and appropriately express *Gata3* (Figure S9). Moreover, type II ganglion fibers innervating the OHCs are unmyelinated, different from type I fibers innervating the IHCs, indicating that the r4-neural crest-derived YFP^+^ Schwann cells myelinize mainly type I fibers. Furthermore, we rule out abnormalities of the second arch-derived middle ear ossicles potentially contributing to the auditory phenotype observed in this study, since they are unaffected in both *Hoxb1* and *Hoxb2* mutant mice [58], [61]. Finally, we also exclude a involvement of LOC efferent neurons, which, even if affected in our mutants, appear to have no direct effect on cochlear thresholds measured by ABR [22].

Although we cannot ascertain the major structure responsible for the increased auditory threshold, we propose that abnormal development of MOC neurons, which are required for proper postnatal survival and functioning of OHCs during the hearing process [28], are involved in the hearing impairments of both mutants. In support of this, the strongest morphological hair cell abnormalities is found towards the apical region of the cochlea, where normally low frequency sounds are perceived [11]. Furthermore, early development of hair cells proceeds normally in the absence of efferent neurons but become affected at later stages when OHCs are dependent on proper MOC innervation [21]. Hence, degeneration of OHCs and consequently, altered hearing thresholds, might be caused by the absence of synaptic/trophic stimulation of cochlear hair cells from the centrifugal OC fibers during a postnatal critical period, which is essential for accurate maturation of OHCs [28]. More support comes from the observations that persistence of some MOC neurons innervating the OHCs in *Hoxb1^lateCKO^* and *Hoxb2^ΔKO^* mutant cochleae is sufficient to partially “rescue” the auditory threshold and OHC morphological defects. This occurs in the presence of seemingly comparable patterning and connectivity defects observed in the CN complex of *Hoxb1* and *Hoxb2* mutants. In addition, no differences in the latencies of the evoked responses are found in our ABR analysis, indicating that the auditory stimuli, when perceived, can travel normally along the successive nuclei of the central auditory pathway, even in the presence of abnormal CN and VLL.

Finally, we found altered hearing thresholds already in one-month-old *Hoxb1^null^* mice that were acoustically isolated at birth, and thus not exposed to noise. This rules out that the observed increased threshold is due to reduced function of the MOC and MEM reflexes, as well as LOC neurons that cannot protect the organ of Corti from noise-induced hearing damage. Nevertheless, this does not exclude that deficiencies in the efferent feedback systems are involved in the progressive age-related degeneration of hearing [76], which is more pronounced in mutant mice than in WT. Hence, the minor increase of threshold with age observed in the *Hoxb1^lateCKO^* compared to *Hoxb1^null^* might be due to the presence of a few efferent neurons in the conditional mice.

Thus, our data suggest that efferent innervations play an important postnatal role in the hearing impairment observed in *Hoxb1* and *Hoxb2* mutants, although we cannot completely rule out that altered development of other auditory structures might also contribute. Selective deletion of MOC efferents during development may further support this hypothesis.

### Selective involvement of individual rhombomeres and *Hox* genes in patterning auditory circuits

Our data unravel a novel function for r4 and its crucial patterning genes, *Hoxb1* and *Hoxb2*, in the ascending sound transmission pathway involving CN and VLL, as well as in the establishment of a sensorimotor reflex circuit formed by PVCN and MOC neurons. We found that within the cochlear nuclear complex, *Hoxa2* expression is primarily maintained in the r2/r3-derived AVCN, whereas *Hoxb2* is highly expressed in r4-derived structures, such as the PVCN, in the r2/r3-derived portion of AVCN and in r3/r5-derived granule cochlear cells. Both genes are expressed in the DCN, which is a r3/r4/r5 derivative ([6], [36] and our study). Such partially overlapping and complementary expression patterns, already observed at early stages, might reflect distinct functional rhombomere-specific pathways within the auditory circuit. In this study we show that the absence of *Hoxa2* mainly affects development of the r2/3-derived AVCN and their respective projections to the contralateral MNTB nucleus, [6], [16]. This phenotype might alter a pathway crucial for sound localization [18]. In contrast, in the absence of Hoxb1 or Hoxb2 functions, PVCN acquires an r3-derived AVCN identity and the resultant projections fail to reach their normal targets, i.e. r4-derived VLL and MOC neurons, which are also affected. As a consequence, the efferent reflexes and the innervation of OHCs by MOC neurons are impaired and *Hoxb1* and *Hoxb2* mutants display hearing problems, although with different severities. Thus, *Hoxb1* and *Hoxb2* appear to act primarily upon r4-derived structures, contributing to the main pathway of sound perception, protection and amplification, whereas *Hoxa2* seems to contribute to the sound localization circuitry centered in r3 and r5. In summary, our data support a model whereby rhombomere-specific (thus A-P) and alar- to basal-restricted pools of neurons (thus D-V) contribute to distinct functional pathways and circuits by maintaining differential expression levels of specific *Hox* gene combinations that, in turn, will continuously refine regional identity within the multi-segmental neuronal columns of the hindbrain (see also Model in Figure 1L, 1M).

## Materials and Methods

### Generation of *Hox* mouse mutant lines and matings

For the detailed generation of the *b1r4-Cre* transgenic line, the *Hoxb1^flox^* and *Hoxb1^null^* mutant mice, and the *Hoxb2^ΔKO^* line see the Protocol S1 section. Generation of the *Hoxa2^null^* and *Hoxa2^flox^* alleles are described in other studies [65], [83]. The *b1r4-Cre* mice were crossed with the ROSA26YY reporter line [43] to obtain double heterozygous *b1r4-Cre/YFP* mice in which r4 and r4-derivatives are selectively labeled. *Hoxb1^flox^* mice were mated to the *b1r4-Cre* or *b1r4-Cre/YFP* transgenic lines to obtain *Hoxb1^lateCKO^* in which *Hoxb1* is inactivated exclusively in r4 at around E9.5. Similarly, *Hoxb1^null^* mice were mated to the *b1r4-Cre/YFP* line to permanently label r4 and r4-derivatives in a null background. All experiments were conducted following guidelines of the Institutional Animal Care and Use Committee of the Cardarelli Hospital, Naples, Italy, the University of Nice Sophia-Antipolis, Nice, France and the Friedrich Miescher Institute, Basel, Switzerland.

### Tissue preparation

Adult and P8 mice were perfused with 4% paraformaldehyde (PFA). Embryos, brains and cochleae were fixed overnight in 4% paraformaldehyde (PFA) in phosphate-buffered saline, pH 7.4 (PBS). Tissues were cryoprotected with 10, 20 and 30% sucrose in PBS and frozen in OCT embedding matrix (Kaltek) and sectioned at 14 µm (E10.5 brains; transversal plane), 16 µm (E10.5 embryos sagittal plane; E14.5 to E18.5 brains), or 20 µm (P8 and adult brains).

### Immunohistochemistry and Nissl staining

After inactivation of endogenous peroxidase with 0.5% H_2_O_2_, tissue cryosections were blocked in blocking buffer (0.05% Tween 20, 20% newborn calf serum, NBCS, in PBS) and incubated overnight at 4°C with primary antibodies diluted in hybridization buffer (0.05% Tween 20, 5% NBCS in PBS): anti-GFP rabbit polyclonal antibody (1∶1500/1∶500, Molecular Probes), anti-ChAT goat polyclonal (1∶100, Chemicon), anti-Islet mouse monoclonal (1∶300 clone 39.4D5; Developmental Studies Hybridoma Bank). Sections were washed in blocking buffer and incubated for an hour and a half at room temperature with secondary antibodies: biotinylated rabbit anti-goat (1∶300), goat anti-rabbit or goat anti-mouse (1∶200). The Vectastain Elite ABC kit and DAB substrate kit for peroxidase (Vector) were used for immunohistochemical staining. Nissl staining of frozen sections was performed using standard procedures and the slides were mounted with EUKITT mounting medium. Embryos/pups for each genotype were tested at least twice with the antibodies listed above.

### Immunofluorescence

Tissue cryosections or whole mount dissected cochleae were incubated overnight at 4°C with primary antibodies diluted in blocking buffer (5% goat serum, 6% BSA, 20 mM MgCl_2_, 0.3% Tween 20 in PBS): rabbit anti-GFP (1∶500, Chemicon), mouse anti-Cre (1∶200, BABCO), rabbit anti-Pax6 (1∶100, Chemicon), rabbit anti-Hoxb1 (1∶200, Covance), rabbit anti-Phox2b (Pattyn et al., 1997) (1∶750, gift from C.Goridis), mouse anti-Gata3 (1∶50, Santa Cruz), mouse anti-Islet (1∶300 clone 39.4D5; Developmental Studies Hybridoma Bank), rabbit anti-calbindin (1∶2500, Swant), rabbit anti-calretinin (1∶3000, Swant), guinea pig anti-Sox10 (1∶1000, gift from M. Wegner [84]), rabbit anti-Atoh1 (1∶500, gift from J. Johnson). For Atoh1 antibody immunofluorescence, a particular blocking buffer was used (1% goat serum, 0.1% Triton-X). Tissue was washed in PBS 0.1% Triton-X and incubated for an hour at room temperature with secondary antibodies: Alexa Fluor 488 (green) goat anti-mouse and goat anti-rabbit, Alexa Fluor 594 (red) goat anti-mouse, goat anti-rabbit and goat anti-guinea pig (1∶400, Molecular Probes). The slides were mounted with the Vectastain elite ABC kit (Vector). Embryos/pups for each genotype were tested at least twice with the antibodies listed above.

### *In situ* hybridization

In situ hybridization on cryosections or whole-mount preparations of embryos, as well as combined in situ hybridization and immunohistochemistry, were performed as previously described [85]. The tissue was hybridized using digoxigenin-labeled (Roche labeling kit) riboprobes for *Gata2*, *Gata3*, *Tbx20*, *VGlut2*, *Gad67*, *Hoxb1*, *Hoxb2*, *Hoxa2*, *Atoh7*, *Atoh1*, *Barhl1*, *Robo3*, *Cre-recombinase*. Embryos/pups for each genotype were tested at least twice with the riboprobes listed above.

### Retrograde tracing from the cochlea

P8 heads of WT (n = 4) and *Hoxb1* mutants (n = 4) were dissected and fixed overnight in 4% PFA. A crystal of fluorescent carbocyanine dye DiI (Molecular Probes) was placed unilaterally in the cochlea (exposed through the base of the cranium) and allowed to diffuse for 1 to 3 months (the first two months at 37°C; afterwards at RT) in PBS containing 0.025% sodium azide. Rhodamine-conjugated dextran (Molecular Probes, Eugene, Oregon) was employed for tracing the MOC, AVCN and PVCN projections of WT (n = 5), *Hoxb2* (n = 3) and *Hoxa2* (n = 2) mutants. E18.5 and P8 heads (MOC tracing) and brains (AVCN and PVCN tracing) were dissected and dextran crystals were inserted unilaterally into cochlea, AVCN and PVCN regions, respectively. The embryos were incubated for 8–12 hours as described in [7]. All brains were fixed in 4% PFA, embedded in 3% agarose in PBS and vibratome-sectioned (100 µm-thick).

### Auditory brainstem response (ABR) and statistical analysis

ABR recordings were performed as previously reported [86] and described in the Protocol S1. The graph plot mean ± SE (standard error) statistics for dual comparisons and were generated using Student's t-tests, whereas statistics for multiple comparisons were generated using one-way analysis of variance (ANOVA) followed by a suitable post hoc t-test; ^*^0.01≤p<0.05, ^**^0.001≤p<0.01, ^***^p<0.001 for all statistics herein.

### Scanning electron microscopy (SEM)

For SEM, cochleae were fixed in 2.5% glutaraldehyde in 0.1 M phosphate-buffered saline (PBS) pH 7.4 (19 ml of 0.2 M sodium phosphate monobasic NaH_2_PO_4_ and 81 ml of 0.2 M sodium phosphate bibasic Na_2_HPO_4_) for 4 h at 4°C and rinsed in PBS over night. The organs of Corti were isolated, rinsed in PBS and post-fixed in 1% OsO_4_ in the same buffer for 1 h at 4°C. After several rinses in PBS, the cochleae were separated in apical and basal turn and then the specimens were subjected to serial dehydration followed by critical point drying. The samples were mounted on aluminum stubs and sputter coated with gold. The processed specimens were investigated and photographed using a JEOL 6700F SEM operated at 5 kV and at a 8.3 mm working distance. SEM images were collected digitally.

### Transmission electron microscopy (TEM)

For TEM, cochleae were fixed in 2.5% glutaraldehyde in 0.1 M phosphate-buffered saline (PBS) pH 7.4 for 4 h at 4°C and rinsed in PBS over night. The organs of Corti were isolated, rinsed in PBS and postfixation was based on 1% osmium tetroxide solution (Fluka) in a 0.05 M PBS at pH 7.4 cooled on ice for 1 h. Specimens were dehydrated with ethanol and then, with propylene oxide and embedded in Epon 812 resin (Fluka). The blocks containing cells were cut using a Super Nova Leica Ultratome. Semithin sections at 2 µm thickness were studied with a light microscope (Polivar Reichert-Jung) after staining with 1% toluidine blue (Carlo Erba). Ultrathin sections (80 nm) were stained with 2% uranyl acetate (Electron Microscopy Sciences) for 10 min at room temperature and 2.66% lead citrate (Electron Microscopy Sciences) for 8 min at room temperature. Grids were examined by using a Philips EM 208 S transmission electron microscope (Philips) operating at 80 kV.

### Quantification and statistical analysis

The VLL size was quantified on adjacent sections of P8 WT, *Hoxb1^null^*, *Hoxb1^lateCKO^* and *Hoxb2^ΔKO^* and of E18.5 WT and *Hoxa2^null^* brainstems hybridized with *Gad67*. The area of the VLL was measured using the MacBiophotonics Image J software. The sum of the area of all sections was expressed as a percentage of the VLL area in wt.

The loss of outer hair cells (OHCs) on SEM sections was quantified on 10 non-overlapping areas of 3060 µ^2^, considering at least one area per sample. Quantitative data are depicted as mean with standard error (SE) of the mean obtained from at least 3 pups or animals tested for significance by the unpaired Student's *t*-test or for multiple comparisons by the one-way analysis of variance (ANOVA) followed by a suitable post hoc t-test; ^*^0.01≤p<0.05, ^**^0.001≤p<0.01, ^***^p<0.001 for all statistics herein.

## Supporting Information

Figure S1 Generation of a novel r4-restricted *Cre* driver line and expression of the *Cre-recombinase*. (A) Schematic diagram of the *b1r4-Cre-recombinase* construct. The gene of the *Cre-recombinase* is cloned downstream of the *Wnt1* basic promoter [87] and under the control of the *Hoxb1* r4 enhancer [42]. (B) Genotyping of the *b1r4-Cre* mice by PCR using internal primers for *Cre* and primers for *actin* as an internal PCR control. (C) Lateral and dorsal views of E8.0 to E8.25 embryos hybridized with *Cre-recombinase*. Note that *Cre-recombinase* starts to be expressed around E8.0 in a patchy way. (D) At E9.0 *Cre* is expressed in all cells and restricted solely to r4.
(TIF)

Figure S2 Dorsal r4-derivatives are complementary to the *Atoh1*- and *Barlh1*-positive rhombic lip regions and contribute to the cochlear nucleus. (A) Schematic of an E10.5 coronal section indicating the position of the rhombic lip region, from which the adjacent pictures are taken. Details of the dorsal region of r4 indicate that Atoh1^+^ cells express YFP, as seen by the merging of the two images. (B) Schematic of an E14.5 coronal section through the rhombic flexure, in which the position of upper and lower rhombic lip cells (URL, LRL) appears colored in brown. The boxed area indicates the region shown in the adjacent panels. Only a few r4/YFP^+^ cells co-localize with *Atoh1*-expressing cells, but no *Barlh1*-expressing cells are positive for YFP, as also seen in high magnification details. (C) Schematics illustrating the position of the cochlear area in a lateral parasagittal plane. The boxed area indicates the magnified region shown in D and E. (D, E). Immunodetection of YFP protein at E14.5 and E16.5 illustrates progressive migration of the r4 lower rhombic lip (LRL)-derived YFP-positive cells to the cochlear nuclear complex (CN). cp, choroid plexus; cb, cerebellum. Scale bars, 20 µm (A), 200 µm in (B), 50 µm insets in (B), 100 µm in (D, E).
(TIF)

Figure S3 Targeting strategy to generate *Hoxb1^floxneo^* embryonic stem (ES) cells. (A) Schematic diagram of the *Hoxb1* locus, the targeting construct and the *Hoxb1^floxneo^* targeted allele. The construct contains a *loxP* site upstream of the r4 enhancer and two *loxP* sites flanking the positive selector, the neomycin gene, in the intron; a negative selector, the Diphtheria Toxin subunit A (DTA), is located downstream of the 3′ homology region. Two heterospecific FRT sites are inserted internal to the two first lox sites; these sites can be used to knock-in any putative target gene into the *Hoxb1* locus with the help of the recombinase-mediated cassette exchange (RMCE) technology [88]. The *Hoxb1^floxneo^* allele was obtained by homologous recombination between the 5′ 2.8 kb EcoRV-ApaI and the 3′ 6.6 kb ScaI-SacI *Hoxb1* genomic regions. (B–E) Identification of *Hoxb1^floxneo^* ES cells by PCR (B, C) and Southern blot (D, E). PCR specific primers (arrows in A) discern wt and recombinant alleles (B) and amplify the three loxP sites (C). Southern blotting confirms proper homologous recombination after digestion genomic DNA with BamHI and using a 5′ internal probe (D), resulting in a 6.7 kb-long fragment for the *wt* allele and a 3.5 kb-long fragment for the recombined *Hoxb1^floxneo^* allele, and after digestion with SmaI-NdeI and using a 3′ internal probe (E) giving 8.0 kb and 9.6 kb fragments for the *wt* and *Hoxb1^floxneo^* alleles, respectively. The probes and the restriction fragments are indicated in A. The asterisks indicate non-homologous recombinant clones. The arrow indicates the clone used to generate *Hoxb1^flox^* and *Hoxb1^null^* ES cells.
(TIF)

Figure S4 Targeting strategy to generate *Hoxb1^flox^* and *Hoxb1^null^* mice. (A) Diagram of *Hoxb1* locus and *Hoxb1^floxneo^*, *Hoxb1^flox^* and *Hoxb1^null^* targeting alleles. To obtain “floxed” and “null” alleles, a *Hoxb1^floxneo^* clone was electroporated with a plasmid expressing *Cre-recombinase*, which excises the regions between the different combinations of two lox sites, generating in this way distinct types of alleles. The *Hoxb1^flox^* allele, obtained by excision of the neomycin (neo) gene, contains the FRT/lox sites flanking the *Hoxb1* genomic region that was conditionally ablated after mating with the r4-*Cre*-*recombinase* line. In the *Hoxb1^null^* allele the region flanked by the FRT/loxP sites is also excised. (B, C) Identification of *Hoxb1^flox^* and *Hoxb1^null^* ES clones by PCR (B) and Southern blot (C). Three different PCR reactions (B) were used to identify the *wt*, *Hoxb1^flox^*, *Hoxb1^null^* alleles. Southern blot analysis (C) confirms the presence of the recombined clones. 2.2 kb- (*wt*), 3.4 kb- (*Hoxb1^null^*), 5.9 kb- (*Hoxb1^flox^*) and 7.5 kb- (*Hoxb1^floxneo^*) long fragments were obtained after ScaI-EcoRV genomic digestion and using an internal probe. The probe and the restriction fragments are indicated in A. (D) Genotyping of *Hoxb1^flox^* and *Hoxb1^null^* mice by PCR to identify wt, homozygous and heterozygous mutant mice.
(TIF)

Figure S5 Abnormal development of olivocochlear (OC) efferent neurons in *Hoxb1* and *Hoxb2* mutant embryos. (A) Lateral view of the E10.5 r4/YFP^+^ embryo; the line indicates the plane of section. To the right, schematic representation of a coronal section illustrating the areas shown in B. (B) No inner ear efferent (IEE) neurons, identified as double Gata3/Phox2b- or double *Gata2*/Isl1-expressing cells, are detected in E10.5 *Hoxb1^null^* and *Hoxb1^lateCKO^* mutant embryos. In addition, the population of Isl1^+^ cells is reduced in both mutants. The asterisks indicate absence of Phox2b in r4 progenitors, as previously described [59]. (C) In ventral r4 of E10.5 *Hoxb2^ΔKO^* embryos, Isl1^+^ visceral motor neurons (vMN) (which include FBM and IEE) and *Gata3^+^* IEE are present, but reduced. (D) Schematic representation of an E14.5 sagittal section indicating the plane of section and the corresponding coronal section. (E) The small group of OC neurons, normally positioned at the r4/r5 margin and positive for YFP, ChAT and *Gata3* cannot be identified in *Hoxb1^null^* and *Hoxb1^lateCKO^* embryos. (F) In contrast, a tiny but compact group of cells positive for *Gata3* and *Tbx20* can be identified in E14.5 *Hoxb2^ΔKO^* embryos, although in a more dorsal location than normal and close to the abnormally positioned “FBM” nucleus, previously described [64]. fp, floor plate; vz, ventricular zone; mz, marginal zone; FBM, facial branchiomotor neurons; VLLn, nucleus of lateral lemniscus. Scale bars, 100 µm (B, C); 200 µm (E, F).
(TIF)

Figure S6 Reduced VLL in *Hoxb1* mutant brains at E18.5. (A) Schematic view of a brain; the red line shows the plane of sections. (B) Adjacent coronal sections of E18.5 WT, *Hoxb1^null^* and *Hoxb1^lateCKO^* mutants stained for Nissl and YFP, and hybridized with *Gata3*, GABAergic/glycinergic *Gad67* and glutamatergic *vGlut2* markers. Only a few YFP^+^ and *Gad67* ^+^ scattered cells are identified in *Hoxb1^null^* VLL at this stage, whereas no *Gata3^+^* (though some cells are recovered at P8) and *vGlut2^+^* neurons are detected (arrows). The reduction of the VLL and relative expression of its markers is less severe in *Hoxb1^lateCKO^* mutants (arrowheads), as also confirmed postnatally (see Figure 3). No ectopic expression of *VGlut2* is detected in the VLL region of mutant mice. The DLL and the PN are not affected. VLL, ventral nucleus of lateral lemniscus; DLL, dorsal nucleus of lateral lemniscus; PN, pontine nucleus; IC inferior colliculus; FBM, facial branchiomotor neurons; MG, medial geniculate nucleus. Scale bars, 200 µm.
(TIF)

Figure S7 Cholinergic LOC neurons are strongly affected in *Hoxb1* mutant brains. (A) Adjacent coronal sections of ventral P8 WT brains stained with the cholinergic marker ChAT and the transcription factor *Tbx20*, labeling LOC and MOC motor neurons (red arrowhead), and the glutamatergic marker *vGlut2* expressed by LSO neurons. The boxes in A indicate the area where the high magnifications of (B) are taken. (B) High magnifications of WT, *Hoxb1^null^* and *Hoxb1^lateCKO^* mutant brains. The cholinergic population is the most severely affected population in *Hoxb1* mutant brains, as seen by complete absence of ChAT- and *Tbx20*-expressing MOC and LOC neurons in *Hoxb1^null^* and the presence of only few LOC neurons in *Hoxb1^lateCKO^* mice (black arrowhead). On the contrary, the glutamatergic population in the LSO is almost preserved, although slightly less *VGlut2^+^* neurons are found, particularly in *Hoxb1^null^*, in the region derived from r4 (shown in Figure 1F). LSO, lateral superior olive nucleus; LOC, lateral olivocochlear neurons; MOC, medial olivocochlear neurons; Vn trigeminal motor nucleus. Scale bars, 400 µm (A), 200 µm (B).
(TIF)

Figure S8 Moderate outer hair cell abnormalities in basal turns of *Hoxb1^null^* cochleae. (A) Scanning electron microscopy (SEM) views of the cochlea at P8: an overview of basal turns of WT, *Hoxb1^null^* and *Hoxb1^lateCKO^* cochleae and representative high magnifications of OHCs. In basal turns the typical V-shaped morphology of OHCs is slightly enlarged compared to their counterparts in the apical turn. Shape and organization of OHCs in the basal cochlear regions are not affected in *Hoxb1* mutants. (B) SEM views of 3-month-old cochleae: an overview of basal turns of WT, *Hoxb1^null^* and *Hoxb1^lateCKO^* and representative high magnifications of OHCs. In *Hoxb1^lateCKO^* and *Hoxb1^null^* cochleae OHCs maintain their regular organization. Slight abnormalities in stereocilia organization and orientation are present only in basal turns of *Hoxb1^null^* (arrows), but not of *Hoxb1^lateCKO^* cochleae. OHCs, outer hair cells; IHCs, inner hair cells. Scale bars, 10 µm (A, B left panels), 1 µm (A, B right panels).
(TIF)

Figure S9 Spiral ganglion glial cells originate from r4. (A) Dissected whole-mount cochleae of WT and *Hoxb1^null^* P8 pups immunostained with an anti-GFP antibody (which cross-reacts with the endogenous YFP) indicate that r4 neural crest cells contribute to glial cells required for myelination of spiral ganglion (spg) neurons and their projections. Note that in *Hoxb1^null^* cochleae YFP^+^ cells are still present although in reduced number. (B) Details of cochleae on adjacent sagittal sections of E18.5 heads immunostained with an anti-GFP antibody and hybridized with *Gata3*, a reliable marker for spiral ganglion neurons and hair cells in the organ of Corti (c) [81]. *Gata3* expression is not changed in spg neurons of *Hoxb1^null^* cochleae despite decreased YFP labeling. This indicates that spiral ganglion neuron differentiation is not affected in the absence of *Hoxb1*. (C) Adjacent sections of (B) immunostained with anti-GFP and the glial lineage marker Sox10 [84]. The merge YFP/Sox10 indicates that YFP^+^ cells express Sox10 in the spiral ganglion (arrowheads in high magnification views of the area indicated in the boxes on the left). A reduced number of double YFP^+^/Sox10^+^ (arrowheads) cells are present in *Hoxb1^null^* mutants. Scale bars, 100 µm (A, C), 200 µm (B).
(TIF)

Protocol S1 (DOC)
