# Supplementary figures and images for: Assembly of the Auditory Circuitry by a Hox Genetic Network in the Mouse Brainstem
Source: PLoS Genet. 2013 Feb 7;9(2):e1003249. doi: 10.1371/journal.pgen.1003249 (PMC3567144; doi:10.1371/journal.pgen.1003249)

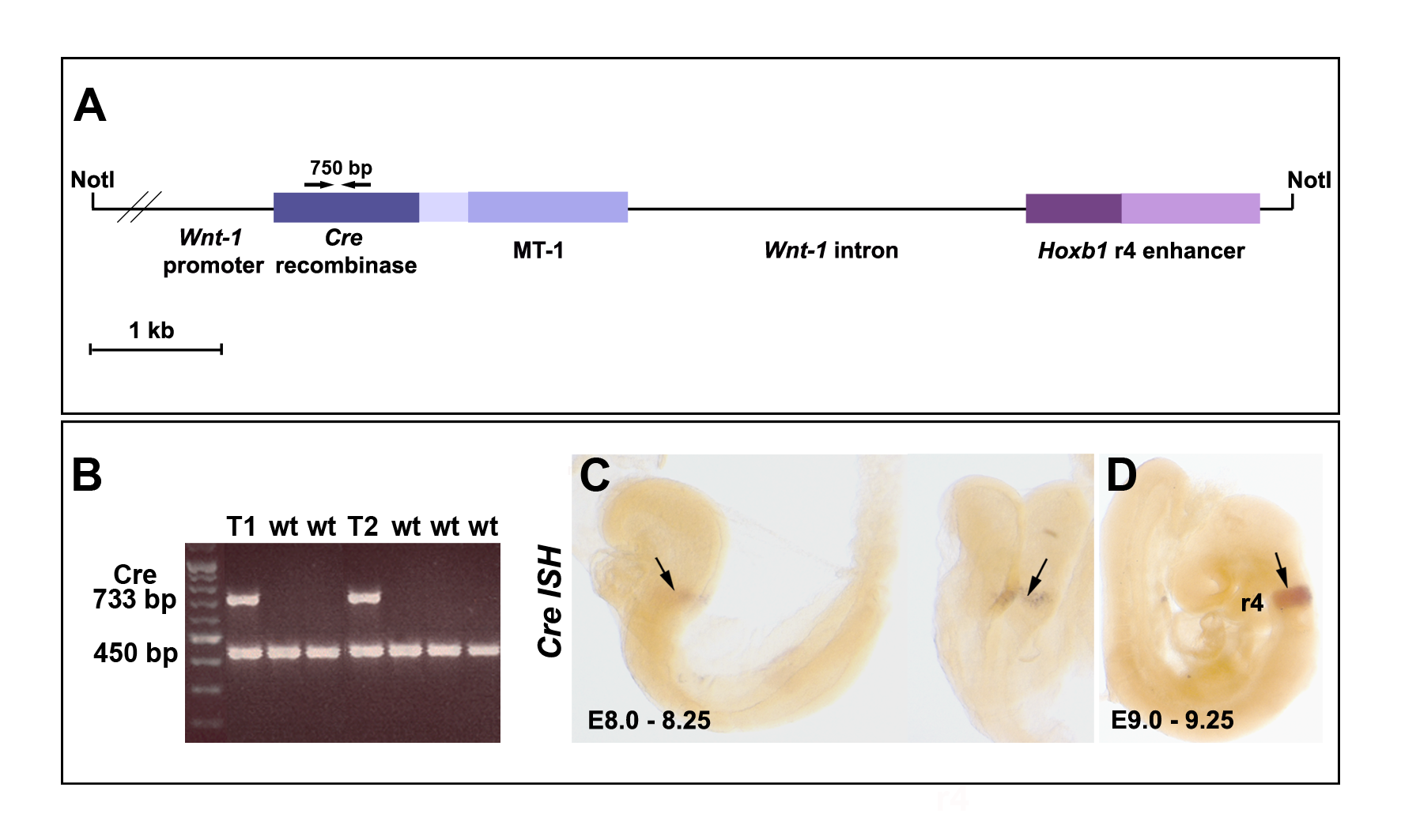

Supplement: Figure S1 — Generation of a novel r4-restricted Cre driver line and expression of the Cre-recombinase. (A) Schematic diagram of the b1r4-Cre-recombinase construct. The gene of the Cre-recombinase is cloned downstream of the Wnt1 basic promoter [87] and under the control of the Hoxb1 r4 enhancer [42]. (B) Genotyping of the b1r4-Cre mice by PCR using internal primers for Cre and primers for actin as an internal PCR control. (C) Lateral and dorsal views of E8.0 to E8.25 embryos hybridized with Cre-recombinase. Note that Cre-recombinase starts to be expressed around E8.0 in a patchy way. (D) At E9.0 Cre is expressed in all cells and restricted solely to r4. (TIF) [file pgen.1003249.s001.tif]

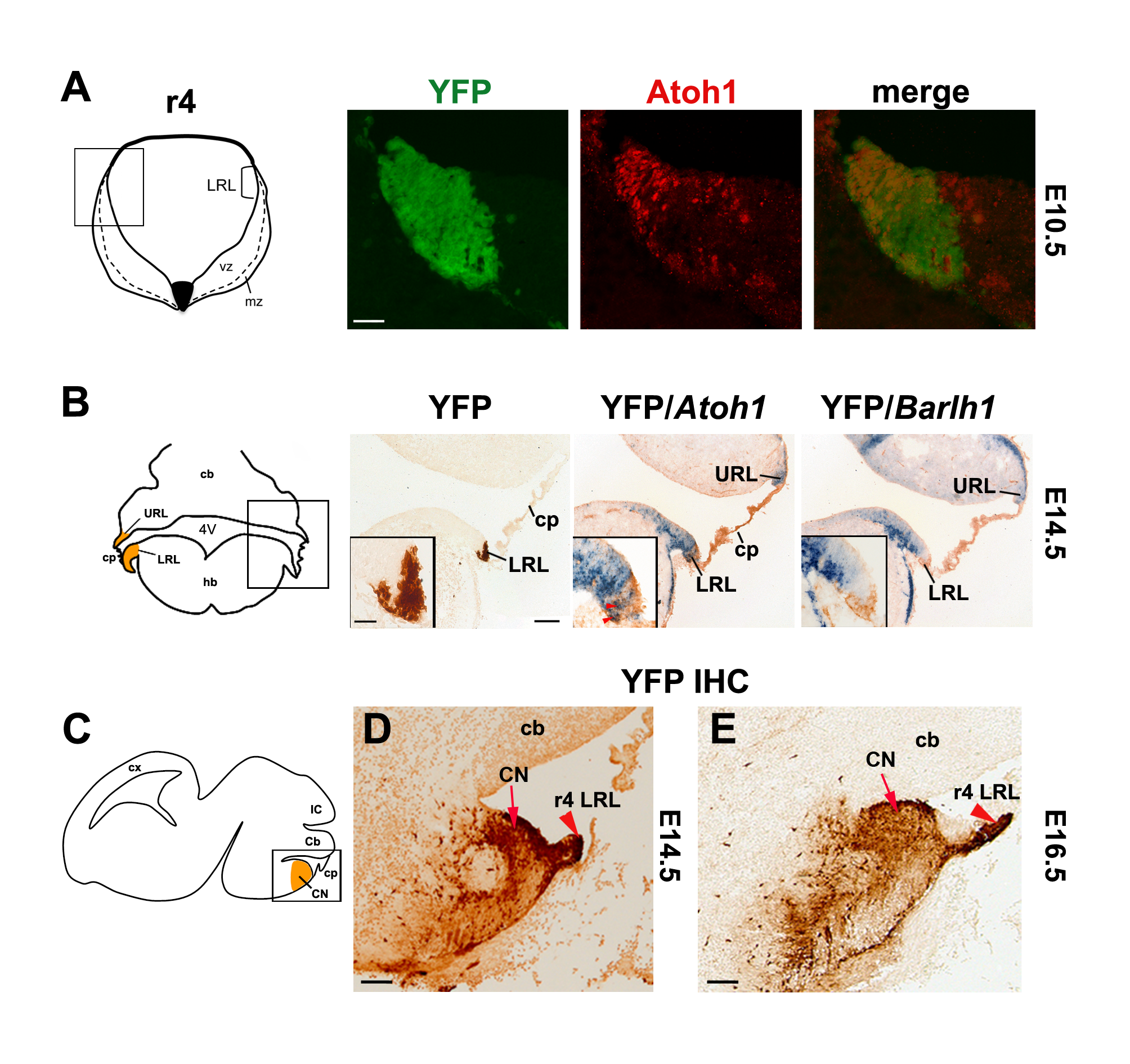

Supplement: Figure S2 — Dorsal r4-derivatives are complementary to the Atoh1- and Barlh1-positive rhombic lip regions and contribute to the cochlear nucleus. (A) Schematic of an E10.5 coronal section indicating the position of the rhombic lip region, from which the adjacent pictures are taken. Details of the dorsal region of r4 indicate that Atoh1+ cells express YFP, as seen by the merging of the two images. (B) Schematic of an E14.5 coronal section through the rhombic flexure, in which the position of upper and lower rhombic lip cells (URL, LRL) appears colored in brown. The boxed area indicates the region shown in the adjacent panels. Only a few r4/YFP+ cells co-localize with Atoh1-expressing cells, but no Barlh1-expressing cells are positive for YFP, as also seen in high magnification details. (C) Schematics illustrating the position of the cochlear area in a lateral parasagittal plane. The boxed area indicates the magnified region shown in D and E. (D, E). Immunodetection of YFP protein at E14.5 and E16.5 illustrates progressive migration of the r4 lower rhombic lip (LRL)-derived YFP-positive cells to the cochlear nuclear complex (CN). cp, choroid plexus; cb, cerebellum. Scale bars, 20 µm (A), 200 µm in (B), 50 µm insets in (B), 100 µm in (D, E). (TIF) [file pgen.1003249.s002.tif]

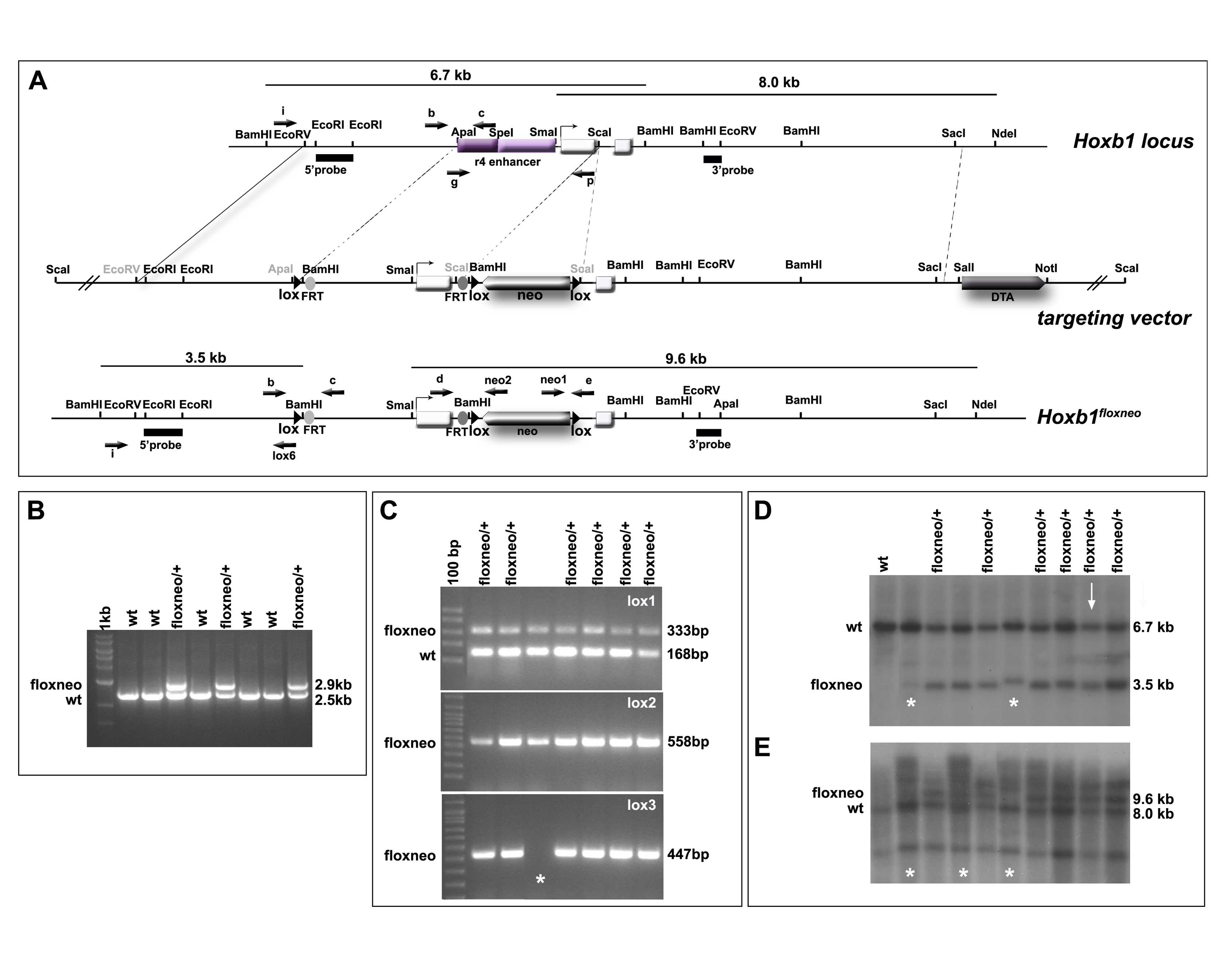

Supplement: Figure S3 — Targeting strategy to generate Hoxb1floxneo embryonic stem (ES) cells. (A) Schematic diagram of the Hoxb1 locus, the targeting construct and the Hoxb1floxneo targeted allele. The construct contains a loxP site upstream of the r4 enhancer and two loxP sites flanking the positive selector, the neomycin gene, in the intron; a negative selector, the Diphtheria Toxin subunit A (DTA), is located downstream of the 3′ homology region. Two heterospecific FRT sites are inserted internal to the two first lox sites; these sites can be used to knock-in any putative target gene into the Hoxb1 locus with the help of the recombinase-mediated cassette exchange (RMCE) technology [88]. The Hoxb1floxneo allele was obtained by homologous recombination between the 5′ 2.8 kb EcoRV-ApaI and the 3′ 6.6 kb ScaI-SacI Hoxb1 genomic regions. (B–E) Identification of Hoxb1floxneo ES cells by PCR (B, C) and Southern blot (D, E). PCR specific primers (arrows in A) discern wt and recombinant alleles (B) and amplify the three loxP sites (C). Southern blotting confirms proper homologous recombination after digestion genomic DNA with BamHI and using a 5′ internal probe (D), resulting in a 6.7 kb-long fragment for the wt allele and a 3.5 kb-long fragment for the recombined Hoxb1floxneo allele, and after digestion with SmaI-NdeI and using a 3′ internal probe (E) giving 8.0 kb and 9.6 kb fragments for the wt and Hoxb1floxneo alleles, respectively. The probes and the restriction fragments are indicated in A. The asterisks indicate non-homologous recombinant clones. The arrow indicates the clone used to generate Hoxb1flox and Hoxb1null ES cells. (TIF) [file pgen.1003249.s003.tif]

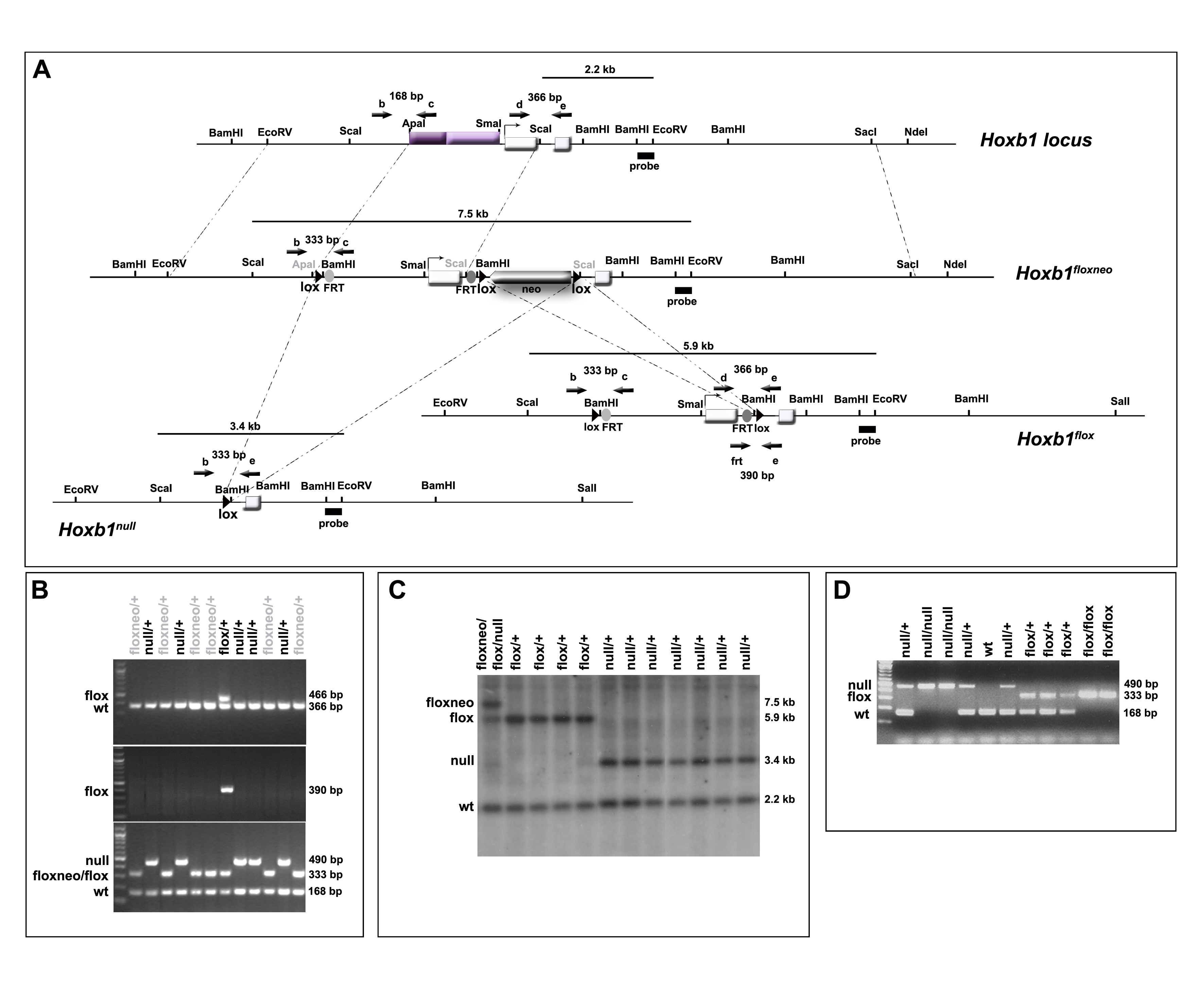

Supplement: Figure S4 — Targeting strategy to generate Hoxb1flox and Hoxb1null mice. (A) Diagram of Hoxb1 locus and Hoxb1floxneo, Hoxb1flox and Hoxb1null targeting alleles. To obtain “floxed” and “null” alleles, a Hoxb1floxneo clone was electroporated with a plasmid expressing Cre-recombinase, which excises the regions between the different combinations of two lox sites, generating in this way distinct types of alleles. The Hoxb1flox allele, obtained by excision of the neomycin (neo) gene, contains the FRT/lox sites flanking the Hoxb1 genomic region that was conditionally ablated after mating with the r4-Cre-recombinase line. In the Hoxb1null allele the region flanked by the FRT/loxP sites is also excised. (B, C) Identification of Hoxb1flox and Hoxb1null ES clones by PCR (B) and Southern blot (C). Three different PCR reactions (B) were used to identify the wt, Hoxb1flox, Hoxb1null alleles. Southern blot analysis (C) confirms the presence of the recombined clones. 2.2 kb- (wt), 3.4 kb- (Hoxb1null), 5.9 kb- (Hoxb1flox) and 7.5 kb- (Hoxb1floxneo) long fragments were obtained after ScaI-EcoRV genomic digestion and using an internal probe. The probe and the restriction fragments are indicated in A. (D) Genotyping of Hoxb1flox and Hoxb1null mice by PCR to identify wt, homozygous and heterozygous mutant mice. (TIF) [file pgen.1003249.s004.tif]

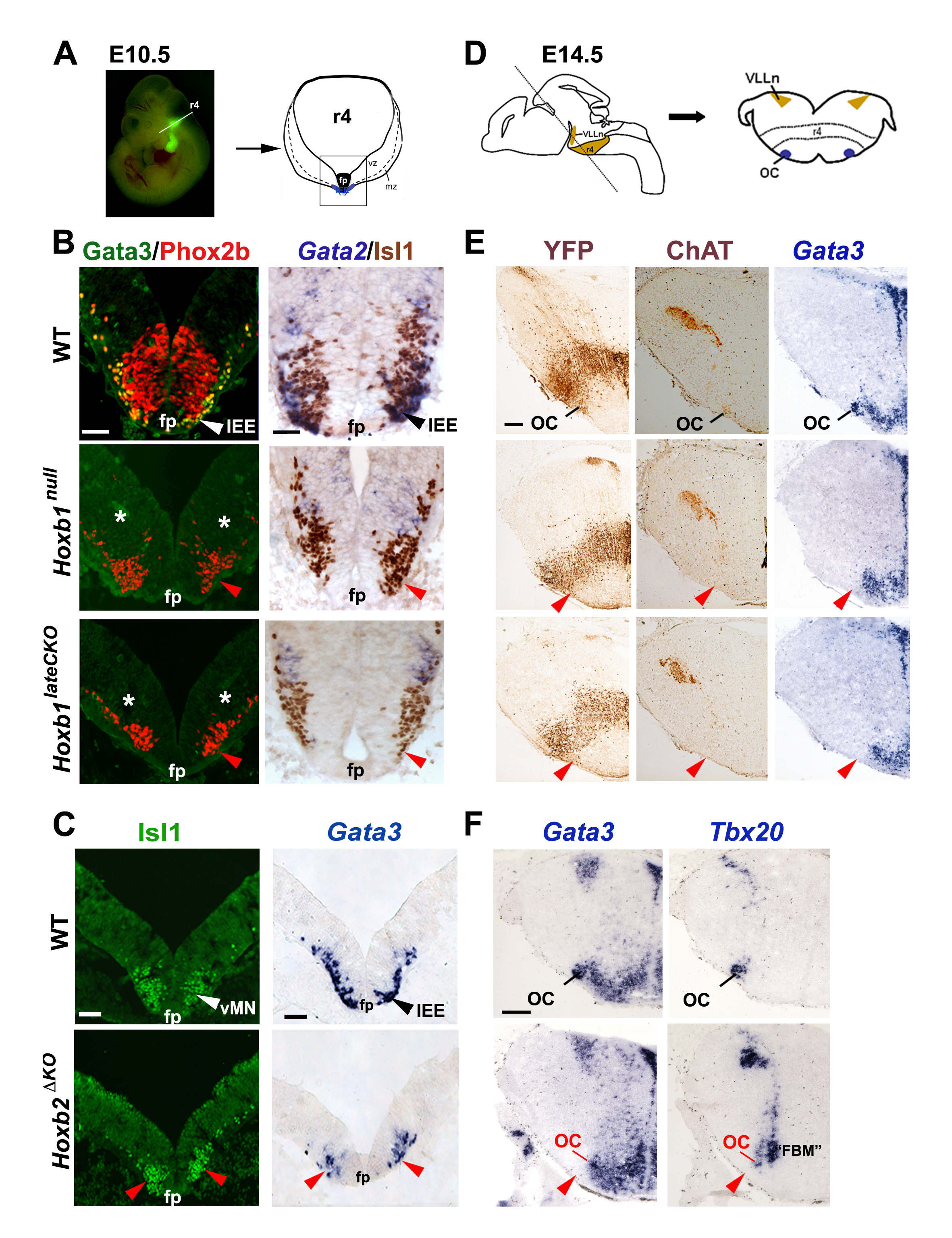

Supplement: Figure S5 — Abnormal development of olivocochlear (OC) efferent neurons in Hoxb1 and Hoxb2 mutant embryos. (A) Lateral view of the E10.5 r4/YFP+ embryo; the line indicates the plane of section. To the right, schematic representation of a coronal section illustrating the areas shown in B. (B) No inner ear efferent (IEE) neurons, identified as double Gata3/Phox2b- or double Gata2/Isl1-expressing cells, are detected in E10.5 Hoxb1null and Hoxb1lateCKO mutant embryos. In addition, the population of Isl1+ cells is reduced in both mutants. The asterisks indicate absence of Phox2b in r4 progenitors, as previously described [59]. (C) In ventral r4 of E10.5 Hoxb2ΔKO embryos, Isl1+ visceral motor neurons (vMN) (which include FBM and IEE) and Gata3+ IEE are present, but reduced. (D) Schematic representation of an E14.5 sagittal section indicating the plane of section and the corresponding coronal section. (E) The small group of OC neurons, normally positioned at the r4/r5 margin and positive for YFP, ChAT and Gata3 cannot be identified in Hoxb1null and Hoxb1lateCKO embryos. (F) In contrast, a tiny but compact group of cells positive for Gata3 and Tbx20 can be identified in E14.5 Hoxb2ΔKO embryos, although in a more dorsal location than normal and close to the abnormally positioned “FBM” nucleus, previously described [64]. fp, floor plate; vz, ventricular zone; mz, marginal zone; FBM, facial branchiomotor neurons; VLLn, nucleus of lateral lemniscus. Scale bars, 100 µm (B, C); 200 µm (E, F). (TIF) [file pgen.1003249.s005.tif]

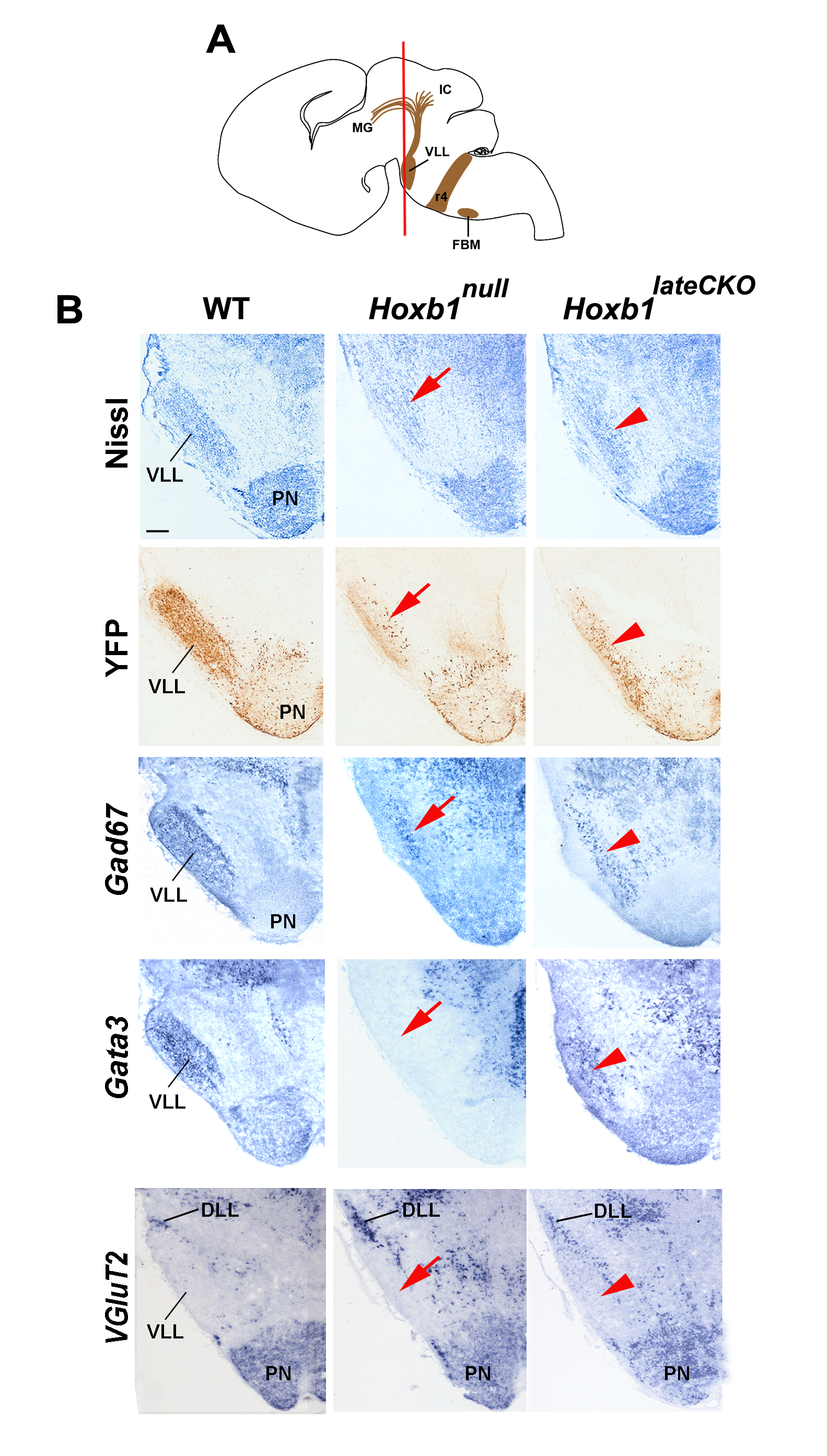

Supplement: Figure S6 — Reduced VLL in Hoxb1 mutant brains at E18.5. (A) Schematic view of a brain; the red line shows the plane of sections. (B) Adjacent coronal sections of E18.5 WT, Hoxb1null and Hoxb1lateCKO mutants stained for Nissl and YFP, and hybridized with Gata3, GABAergic/glycinergic Gad67 and glutamatergic vGlut2 markers. Only a few YFP+ and Gad67 + scattered cells are identified in Hoxb1null VLL at this stage, whereas no Gata3+ (though some cells are recovered at P8) and vGlut2+ neurons are detected (arrows). The reduction of the VLL and relative expression of its markers is less severe in Hoxb1lateCKO mutants (arrowheads), as also confirmed postnatally (see Figure 3). No ectopic expression of VGlut2 is detected in the VLL region of mutant mice. The DLL and the PN are not affected. VLL, ventral nucleus of lateral lemniscus; DLL, dorsal nucleus of lateral lemniscus; PN, pontine nucleus; IC inferior colliculus; FBM, facial branchiomotor neurons; MG, medial geniculate nucleus. Scale bars, 200 µm. (TIF) [file pgen.1003249.s006.tif]

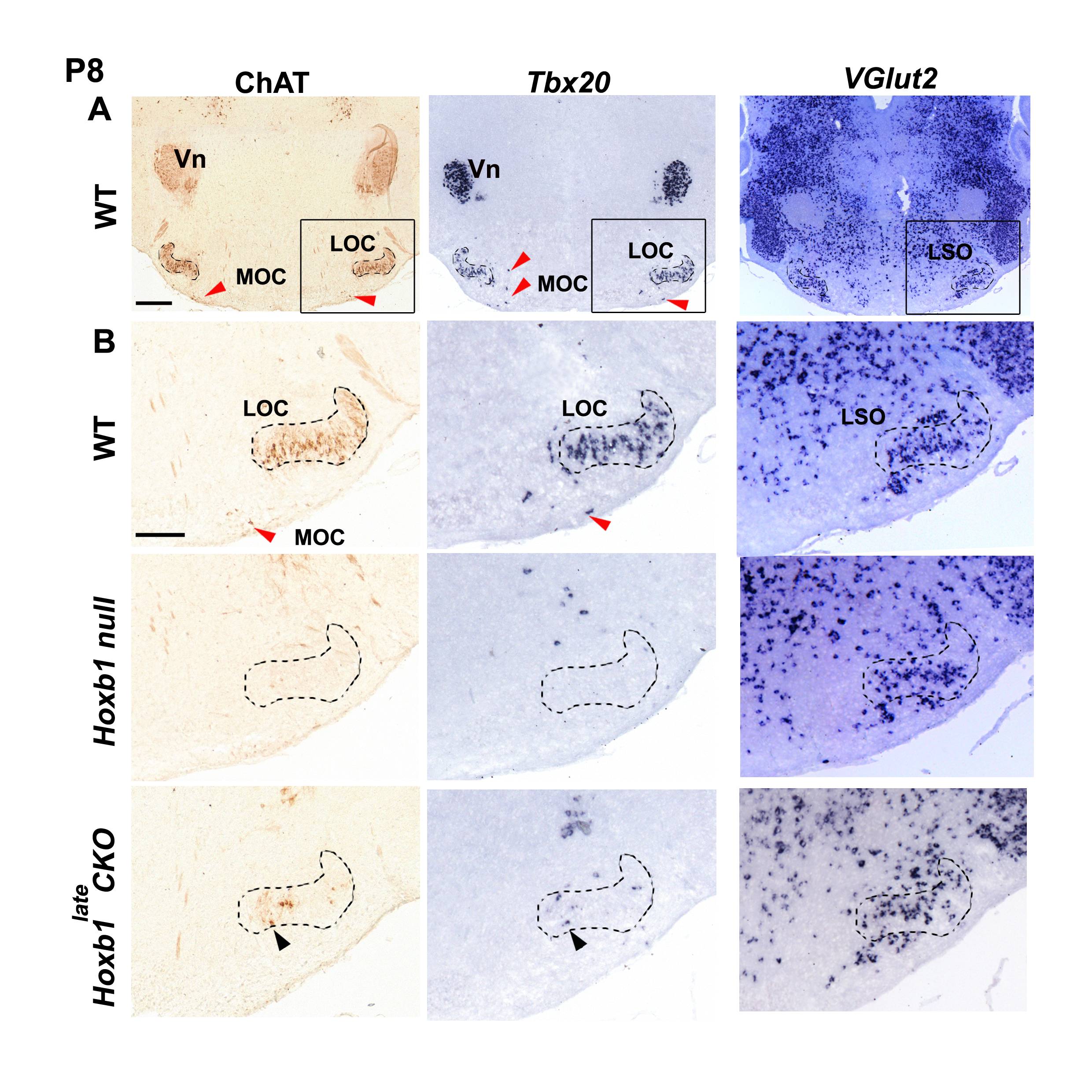

Supplement: Figure S7 — Cholinergic LOC neurons are strongly affected in Hoxb1 mutant brains. (A) Adjacent coronal sections of ventral P8 WT brains stained with the cholinergic marker ChAT and the transcription factor Tbx20, labeling LOC and MOC motor neurons (red arrowhead), and the glutamatergic marker vGlut2 expressed by LSO neurons. The boxes in A indicate the area where the high magnifications of (B) are taken. (B) High magnifications of WT, Hoxb1null and Hoxb1lateCKO mutant brains. The cholinergic population is the most severely affected population in Hoxb1 mutant brains, as seen by complete absence of ChAT- and Tbx20-expressing MOC and LOC neurons in Hoxb1null and the presence of only few LOC neurons in Hoxb1lateCKO mice (black arrowhead). On the contrary, the glutamatergic population in the LSO is almost preserved, although slightly less VGlut2+ neurons are found, particularly in Hoxb1null, in the region derived from r4 (shown in Figure 1F). LSO, lateral superior olive nucleus; LOC, lateral olivocochlear neurons; MOC, medial olivocochlear neurons; Vn trigeminal motor nucleus. Scale bars, 400 µm (A), 200 µm (B). (TIF) [file pgen.1003249.s007.tif]

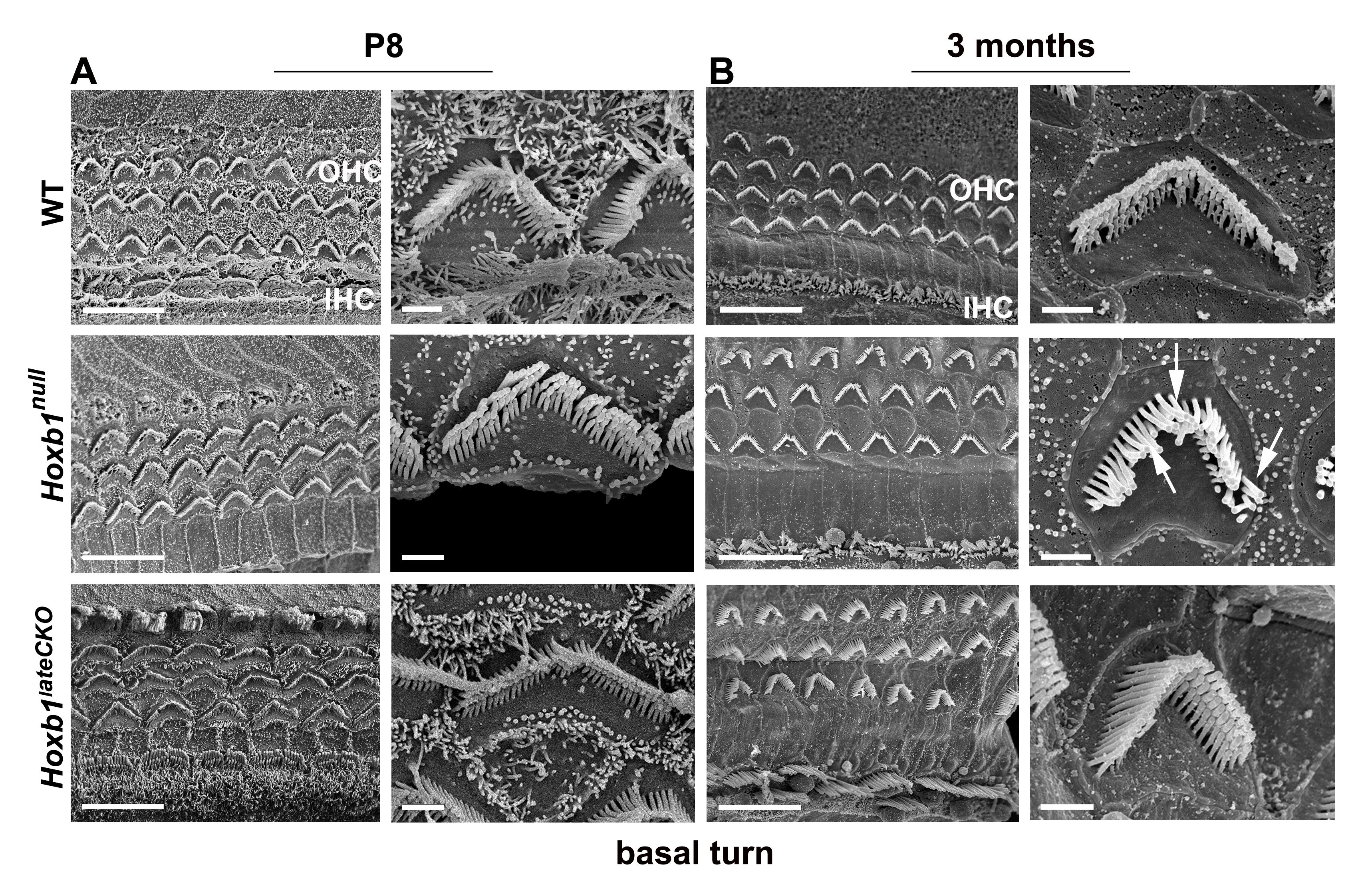

Supplement: Figure S8 — Moderate outer hair cell abnormalities in basal turns of Hoxb1null cochleae. (A) Scanning electron microscopy (SEM) views of the cochlea at P8: an overview of basal turns of WT, Hoxb1null and Hoxb1lateCKO cochleae and representative high magnifications of OHCs. In basal turns the typical V-shaped morphology of OHCs is slightly enlarged compared to their counterparts in the apical turn. Shape and organization of OHCs in the basal cochlear regions are not affected in Hoxb1 mutants. (B) SEM views of 3-month-old cochleae: an overview of basal turns of WT, Hoxb1null and Hoxb1lateCKO and representative high magnifications of OHCs. In Hoxb1lateCKO and Hoxb1null cochleae OHCs maintain their regular organization. Slight abnormalities in stereocilia organization and orientation are present only in basal turns of Hoxb1null (arrows), but not of Hoxb1lateCKO cochleae. OHCs, outer hair cells; IHCs, inner hair cells. Scale bars, 10 µm (A, B left panels), 1 µm (A, B right panels). (TIF) [file pgen.1003249.s008.tif]

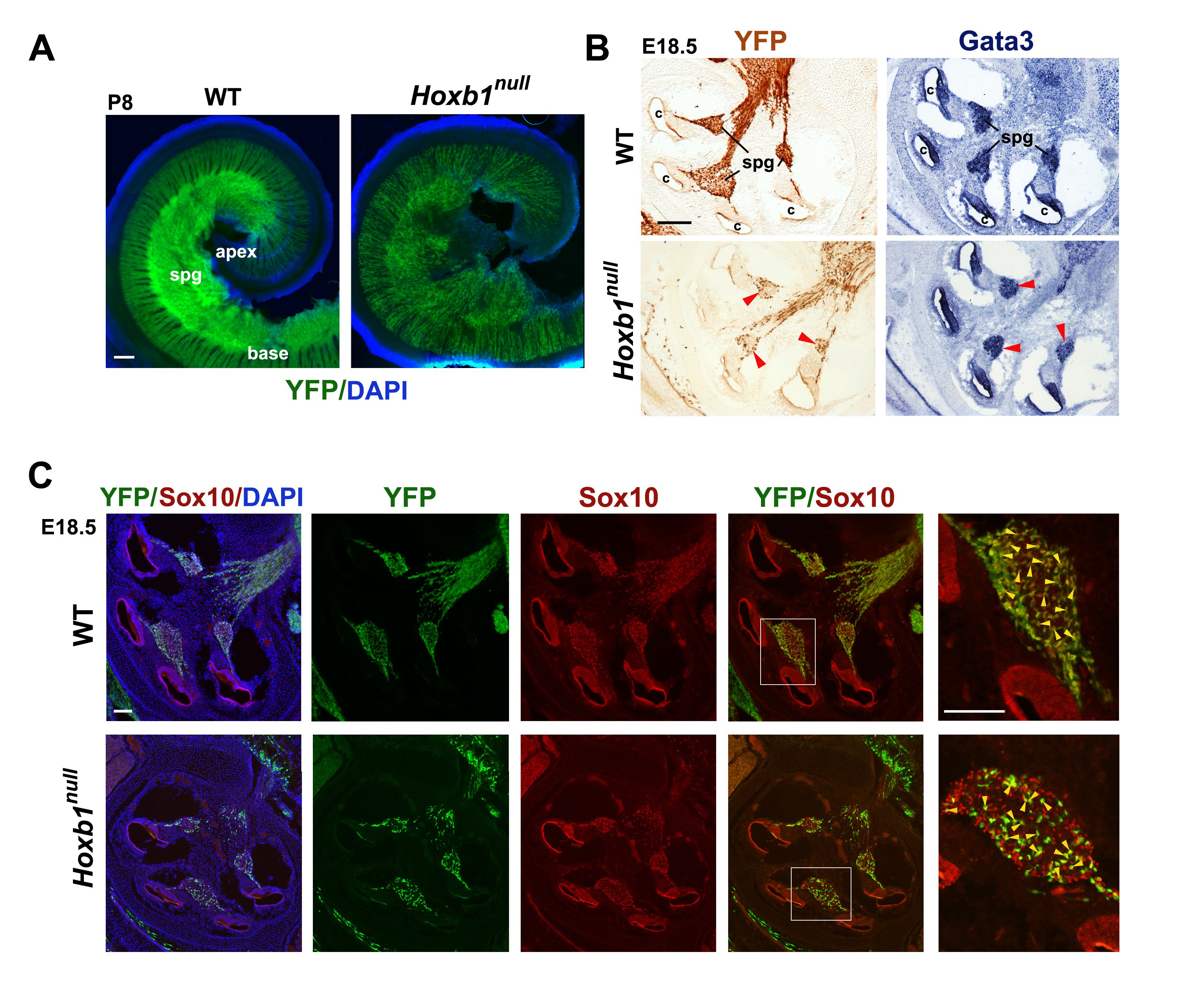

Supplement: Figure S9 — Spiral ganglion glial cells originate from r4. (A) Dissected whole-mount cochleae of WT and Hoxb1null P8 pups immunostained with an anti-GFP antibody (which cross-reacts with the endogenous YFP) indicate that r4 neural crest cells contribute to glial cells required for myelination of spiral ganglion (spg) neurons and their projections. Note that in Hoxb1null cochleae YFP+ cells are still present although in reduced number. (B) Details of cochleae on adjacent sagittal sections of E18.5 heads immunostained with an anti-GFP antibody and hybridized with Gata3, a reliable marker for spiral ganglion neurons and hair cells in the organ of Corti (c) [81]. Gata3 expression is not changed in spg neurons of Hoxb1null cochleae despite decreased YFP labeling. This indicates that spiral ganglion neuron differentiation is not affected in the absence of Hoxb1. (C) Adjacent sections of (B) immunostained with anti-GFP and the glial lineage marker Sox10 [84]. The merge YFP/Sox10 indicates that YFP+ cells express Sox10 in the spiral ganglion (arrowheads in high magnification views of the area indicated in the boxes on the left). A reduced number of double YFP+/Sox10+ (arrowheads) cells are present in Hoxb1null mutants. Scale bars, 100 µm (A, C), 200 µm (B). (TIF) [file pgen.1003249.s009.tif]
