# Supplementary material for: Assembly of the Auditory Circuitry by a Hox Genetic Network in the Mouse Brainstem
Source: PLoS Genet. 2013 Feb 7;9(2):e1003249. doi: 10.1371/journal.pgen.1003249 (PMC3567144; doi:10.1371/journal.pgen.1003249)
Supplement: Protocol S1 — (DOC) [file pgen.1003249.s010.doc]

**Protocol S1**

**Generation of the *b1r4-Cre* transgenic line**

A 2.7 kb XhoI-HindIII fragment containing the *Cre-recombinase* gene and metallothionein-1 polyadenylation signal obtained from pBS185 (GibcoBRL, Cat*#* 10347-011) was cloned into EcoRV-SacII of pWexP3β [1] (gift from Paul S. Danielian). The r4 enhancer/repressor fragment, described previously [2], was inserted in BglII of the same plasmid. The targeting construct was digested with NotI and injected into pronuclei of CD1 oocytes. Genotyping of transgenic mice was performed by PCR using two internal primers for the Cre-recombinase gene,

Cre3 5’-TTGCCGGTCGTGGGCGGCATGGTGC-3’

Cre4 5’-CACGGGCACTGTGTCCAGACCAGGC-3’

that amplified a 733 bp fragment; plus two primers for the housekeeping gene actin,

CCR5mL 5’-CAACCGAGACCTTCCTGTTC-3’

CCR5mR 5’-ATGTGGATGGAGAGGAGTCG-3’

which amplified a 250 bp fragment as an internal PCR control. Two transgenic *b1r4-Cre* mice lines were obtained.

**Generation of *Hoxb1^flox^* and *Hoxb1^null^* mice**.

***Construction of Hoxb1 targeting construct****.* The pFlox vector (gift from D. Chui), containing three lox sites, the neomicine (neo) and timidine kinase (TK) genes, was modified: the BstXI, HindIII, EcoRV restriction sites and the TK gene were eliminated, while a new EcoRV was created upstream of the first lox site. A fragment containing a BamHI-FRTwt -HpaI -FRTF5-BamHI was obtained by annealing two primers:

Frt1:5’GATCCGAAGTTCCTATTCCGAAGTTCCTATTCTCTAGAAAGTATAGGAACTTCGTTAACGAAGTTCCTATTCCGAAGTTCCTATTCTTCAAAAGGTATAGGAACTTCG 3’;

Frt2:3’GCTTCAAGGATAAGGCTTCAAGGATAAGAGATCTTTCATATCCTTGAAGCAATTGCTTCAAGGATAAGGCTTCAAGGATAAGAAGTTTTCCATATCCTTGAAGCCTAG5’ (restriction enzyme sites are underlined; the FRT consensus sites [3] are in red). The fragment obtained was cloned into the BamHI site of pFlox. The 5’ homology arm, a 2.8 kb EcoRV-ApaI genomic region of *Hoxb1*, was inserted into XhoI-KpnI; the 2.5 kb ApaI-ScaI fragment, containing the r4 enhancer of *Hoxb1* and the coding region of the first exon, was inserted into HpaI between two FRT/lox sites and the 3’ homology arm was a 6.6 kb ScaI-SacI fragment cloned into EcoRV-SalI. The negative selector, the A subunit of Diphtheria Toxin (DTA), was added downstream of the 3’ homology region in NotI-SalI.

***Generation of Hoxb1^floxneo^ cells.*** The targeting vector was linearized with XhoI and electoporated into TBV2 cells. 600 clones were isolated and the homologous recombination in the *Hoxb1* locus was identified by PCR using two specific primers for the wild-type allele (sense primer *Hoxb1g* 5’-GACAACTTTGGGCCCTTGAAG-3’ and antisense primer *Hoxb1p* 5’-CTCTTGACCTTCATCCAGTCG-3’), which amplified a 2.5 kb wt fragment, and two specific primers for a recombinant allele (sense primer *Hoxb1i* 5’-CTGTCTAAATAGGGCCAACCCTCTC-3’ and antisense primer lox6 5’-TCCCCGGGCGAGCTCGAATTGATCC-3’), which amplified a 2,9 kb fragment.

PCR was also performed with primers flanking the three lox sites:

*-Hoxb1b* (5’-CTAGAGAGCCAGACAAGGATG-3’) and *Hoxb1c* (5’-AACTTCTCAGGTTTCCTTACC-3’), which amplified a 333 bp lox1 fragment and a 168 bp wt fragment;

-Hoxb1d (5’-CCTTGGCTCGGGCATACACAG-3’) and *neo2* (5’-TCCCGATTCGCAGCGCATCG-3’) amplified a 558 bp lox2 fragment;

-neo1 (5’-CCAGAGGCCACTTGTGTAGC-3)’ and *Hoxb1e* (5’-AGCCACGTCAGTTCTCTCACC-3’) amplified a 447 bp lox3 fragment.

Positive clones were then verified by Southern blotting using BamHI to digest the genomic DNA and a 663 bp EcoRI-EcoRI fragment as 5’ internal probe, obtaining the 6.7 kb wt and 3.5 kb *Hoxb1^floxneo^* fragments. 3’ Southern was performed with SmaI-NdeI using the 3’ internal probe, a 348 bp BamHI-EcoRV fragment, obtaining the 8.0 kb wt and 9.6 kb *Hoxb1^floxneo^* fragments. A total of six clones showed the correct homologous recombination.

***Generation of Hoxb1^flox^ and Hoxb1^null^ mice.*** A *Hoxb1^floxneo^* clone (arrow in Figure S3D) with a correct karyotype was electroporated with the pMCCrePuro (gift from P. Orban) plasmid expressing *Cre-recombinase* to obtain *Hoxb1^flox^* alleles by the excision of the neomycin gene, and *Hoxb1^null^* alleles by excision of the floxed *Hoxb1* sequence. 600 clones were isolated and PCR analysis was used to identify the mutant alleles: using the *Hoxb1d* and *Hoxb1e* primers, 366 bp wt and 466 bp *Hoxb1^flox^* fragments were obtained; with *Hoxb1frt* (5’-GGCAGGTAAGTAACGAAGTTC-3’) and *Hoxb1e* primers a 390 bp *Hoxb1^flox^* fragment was amplified; with *Hoxb1b*-*Hoxb1c* and *Hoxb1b-Hoxb1e* primers 168 bp wt, 333 bp *Hoxb1^floxneo^* or *Hoxb1^flox^* and 490 bp *Hoxb1^null^* fragments were obtained. Four *Hoxb1^flox^* clones and 107 *Hoxb1^null^* clones were identified. For Southern blot analysis, genomic DNA was digested with ScaI-EcoRV and hybridized with the internal probe 348 bp BamHI-EcoRV fragment, obtaining the 2.2 kb wt*,* 3.4 kb *Hoxb1^null^,* 5.9 kb *Hoxb1^flox^* and 7.5 kb *Hoxb1^floxneo^*. Two independent *Hoxb1^flox^* and one *Hoxb1^null^* clones were used for blastocyst injection and gave germline transmission. Genotyping of *Hoxb1* homozygous, heterozygous and wt mice was performed by PCR, using the primers *Hoxb1b*, *Hoxb1c* and *Hoxb1e*, which amplify fragments of 168 bp for wt, 333 bp for *Hoxb1^flox^* and 490 bp for *Hoxb1^null^*, respectively.

**Generation of the *Hoxb2^∆KO^* mutant allele.** A *neo*-cassette driven by the phosphoglycerate kinase (PGK) was inserted in the *Hoxb2* intron, 950 bp downstream of the translational start codon. The PGK-*neo* was flanked by two *Frt* sites for Flp-mediated excision and a *loxP* site immediately following the 3’ *Frt* site. A second *loxP* site was also introduced in the *Hoxb2* 3’UTR, 468 bp downstream of the *Hoxb2* translational stop codon, for Cre-mediated conditional deletion. Thus, neither the *loxP* sites nor the *Frt*-flanked PGK-*neo* cassette interrupted the *Hoxb2* coding sequence. Homologous recombination of the targeting vector was obtained in ES cells and confirmed by Southern blot, PCR, and sequencing analysis. Germline transmission of the *Hoxb2^(Frt-neo-Frt)lox^* allele was obtained, and heterozygous mutant mice were viable and fertile. Flp-mediated excision of the PGK-*neo* cassette was obtained by mating *Hoxb2^(Frt-neo-Frt)lox^* mice to the ACTB:FLPe deleter [4], thus generating the *Hoxb2^flox^*^/^*^+^* allele. *Hoxb2^flox^*^/^*^+^* and *Hoxb2^flox^*^/^*^flox^* mutant mice were produced at Mendelian frequency, had no obvious defects, proved to be fertile, and survived a normal lifespan. The *Hoxb2^∆KO^* allele was then obtained by crossing *Hoxb2^flox^*^/^*^+^* mice with the *CMV::Cre* deleter [5].

**Auditory Brainstem Response (ABR) measurement**

Control and mutant animals were sedated with medetomidine chlorhydrate (1mg/kg) and ketamine (100mg/kg) via intraperitoneal injection, and sedation was reversed with *atipamezole* (medetomidin antagonist) (1mg/kg) after the ABR measurement. Stimulation, averaging and analysis were carried out using an Amplaid MK12 system (Amplifon, Italy). Monaural stimuli consisted of alternating clicks lasting 0,1 ms at a repetition rate of 11/s. ABRs were recorded in both ears via subcutaneously placed tungsten needle electrodes (diameter 0,25mm). The active electrode was inserted in the vertex, the reference electrode was inserted in the posterior bulla region of the left or right ear, and the ground electrode was inserted in the lower back. Ipsilateral stimuli and contralateral masking white noise were presented with ear probes (Madsen Electronics, Kobenhavn, Denmark). The controlateral masker level was set at 50dB HL, while the click level was set at 110 dB SPL and was decreased in 10dB steps until the detection threshold was approached. Filter settings ranged from 100Hz to 2.5kHz. The analysis time was of 12ms, the amplifier sensibility was of 5nV/division and potentials were amplified 10^5^ X. The ABR signals were calibrated as specified by ISO (International Organisation for Standardisation) 389 and ANSI (American National Standards Institute) S3.6 directives. The detection of the threshold was defined on the basis of 2-3 repeated measurements close to threshold. A threshold value is obtained by the mean between the lowest intensity at which the response is revealed and the highest intensity at which a response is absent. The ABR patterns were examined by two independent observers and accepted if both observers agreed. In the mouse, the sequence of five positive ABR waves (PI-PV) are localized to: the cochlea and/or the compound action potential of the VIII nerve (PI), the cochlear nuclei (PII), the contralateral superior olive complex or SOC (PIII), the lateral lemniscus complex (PIV), and the contralateral inferior colliculus or IC (PV).

**References:**

1. Danielian PS, Echelard Y, Vassileva G, McMahon AP (1997) A 5.5-kb enhancer is both necessary and sufficient for regulation of Wnt-1 transcription in vivo. Dev Biol 192: 300-309.

2. Studer M, Popperl H, Marshall H, Kuroiwa A, Krumlauf R (1994) Role of a conserved retinoic acid response element in rhombomere restriction of Hoxb-1. Science 265: 1728-1732.

3. Bode J, Schlake T, Iber M, Schubeler D, Seibler J, et al. (2000) The transgeneticist's toolbox: novel methods for the targeted modification of eukaryotic genomes. Biol Chem 381: 801-813.

4. Rodriguez CI, Buchholz F, Galloway J, Sequerra R, Kasper J, et al. (2000) High-efficiency deleter mice show that FLPe is an alternative to Cre-loxP. Nat Genet 25: 139-140.

5. Dupe V, Davenne M, Brocard J, Dolle P, Mark M, et al. (1997) In vivo functional analysis of the Hoxa-1 3' retinoic acid response element (3'RARE). Development 124: 399-410.
